# LncRNA *Wee1-AS* coordinates oxidative fatty acid metabolism through the activation of mitochondrial CDK1/CYCLIN B1

**DOI:** 10.1038/s41392-025-02558-4

**Published:** 2026-01-10

**Authors:** Hyeon-Ji Kim, Cheolhee Jeong, Sang-Heon Lee, Seungchan An, Gyu Hwan Hyun, Ga Young Lim, Ju-Yeon Kim, Junhyeong Lee, Min-Jung Park, Sung Won Kwon, Won Kim, Minsoo Noh, Yong-Hyun Han, Mi-Ock Lee

**Affiliations:** 1 College of Pharmacy, Seoul National University, Seoul, Korea; 2 Research Institute of Pharmaceutical Sciences, Seoul National University, Seoul, Korea; 3 Department of Veterinary Physiology, College of Veterinary Medicine, Chonnam National University, Gwangju, Korea; 4 College of Veterinary Medicine and BK21 FOUR Program, Chonnam National University, Gwangju, Korea; 5 Department of Internal Medicine, Seoul National University College of Medicine, Seoul Metropolitan Government Boramae Medical Center, Seoul, Korea; 6 Laboratory of Pathology and Physiology, College of Pharmacy, Kangwon National University, Chuncheon, South Korea; 7 Bio-MAX Institute, Seoul National University, Seoul, Korea; 8 Natural Product Research Institute, Seoul National University, Seoul, Korea

**Keywords:** Cell biology, Non-coding RNAs, Physiology

## Abstract

Metabolic dysfunction-associated steatotic liver disease (MASLD) is steadily increasing with life-threatening complications, underscoring the need for new therapeutic targets. In this study, we identified a novel long noncoding RNA, *Wee1-AS*, which is transcribed from the antisense strand of the *Wee1* gene locus. The expression of *Wee1-AS* was greater in hepatocytes, particularly in the region around the central vein, and it was induced in response to high-fat diet challenge. Adeno-associated virus-mediated overexpression of *Wee1-AS* in mice strongly suppressed the symptoms of MASLD, underscoring its pivotal roles. Mechanistically, *Wee1-AS* enhances mitochondrial fatty acid oxidation by activating the CDK1/CYCLIN B1 complex through two mechanisms. First, it suppressed the transcription of the *Wee1* gene by preventing access to the transcriptional machinery. Second, *Wee1-AS* bound and stabilized the CYCLIN B1 protein by suppressing ubiquitin/proteasome-mediated degradation. Notably, treatment with the WEE1 inhibitor adavosertib ameliorated MASLD symptoms by improving mitochondrial function in the liver. Consistently, knockdown of *Wee1-AS* led to lipid accumulation and mitochondrial dysfunction, both of which were reversed by adavosertib treatment in hepatocytes, indicating a functional interplay between *Wee1-AS* and WEE1 in regulating fatty acid oxidation. Furthermore, we identified a human homolog, *LNC106435.1*, which improved mitochondrial function, suggesting that the modulation of *LNC106435.1* may have potential therapeutic implications for managing MASLD.

## Introduction

Metabolic dysfunction-associated steatotic liver disease (MASLD), a new term replacing “nonalcoholic fatty liver disease (NAFLD)”, is the most widespread chronic liver disease and poses an increasingly significant burden on global health care.^1,2^ It encompasses a spectrum spanning from steatosis to metabolic dysfunction-associated steatohepatitis (MASH), a progressive state marked by elevated oxidative stress and lipotoxicity that drive hepatocellular injury and inflammation.^3^ Considering the elevated incidence of MASH and its associated life-threatening complications such as cardiovascular disease and cirrhosis, there has been a significant endeavor to develop effective treatments.^4^ Recently, Resmetirom, an oral liver-targeted thyroid hormone receptor β selective agonist, was approved by the FDA as the first medication for the treatment of MASH.^5^ Additionally, promising outcomes have emerged from phase 3 clinical trials, specifically regarding the resolution of MASH and improvement of fibrosis with the oral pan-peroxisome proliferator-activated receptor agonist, lanifibranor, and the subcutaneous glucagon-like peptide 1 receptor agonist, semaglutide.^6^ However, the multifaceted etiology of MASLD and the presence of compensatory mechanisms through redundant pathways highlight the ongoing necessity for the exploration of new effective molecular targets for therapeutic interventions.

A long noncoding RNA (lncRNA) refers to an RNA molecule exceeding 200 nucleotides in length, that underges transcription but lacks the capacity to encode proteins. On the basis of their genomic position in relation to protein-coding genes, lncRNAs can be categorized into sense, antisense, bidirectional, intronic, intergenic, or enhancer lncRNAs, and their functionalities tend to be subject to their respective positions.^7^ LncRNAs function in the regulation of chromatin remodeling, transcription, RNA stability, translation, protein stability, and protein localization.^8^ LncRNAs display tissue-specific expression profiles and are often dysregulatted in various metabolic diseases including MASLD.^9,10^ For example, the expression of the lncRNA *NEAT1* elevated in the livers of animal models of MASLD and its function is associated with mitochondrial dynamics and lipogenic gene regulation.^11,12^ The lncRNA *MALAT1* is upregulated in patients with MASLD, and its depletion in mouse models improved insulin resistance and hepatic lipid levels, potentially through PPARα upregulation.^13^ Owing to the significant impact of lncRNAs on the pathogenesis of human diseases, several clinical trials have focused on targeted gene regulation via lncRNAs for the treatment of diseases such as lung cancer and acute ischemic stroke.^14^ However, no such trials have been carried out for the treatment of MASLD thus far.

Mitochondria are vital for various cellular functions including energy production via oxidative phosphorylation, maintenance of redox homeostasis, and generation of biosynthetic precursors for cell growth and proliferation.^15^ Progressive malfunction of mitochondria is caused during the progression of MASH due to a sustained supply of fatty acids, which leads to an increase in oxidative stress, and gradual exhaustion of antioxidative capacity.^16,17^ Conserved cell growth and division signaling pathways play crucial roles in mitochondrial bioenergetics.^18^ For example, key components of the cell cycle oscillator, Cdc28p, and its mammalian homolog CDK1, were demonstrated to regulate mitochondrial function by phosphorylating Tom6p, thereby promoting the assembly of the mitochondrial protein import complex.^19^ CDK1 and CYCLIN B1 are translocated into the mitochondrial matrix where they augment mitochondrial function through the phosphorylation of subunits of mitochondrial complex I at G2/M transition.^20^ In addition, CDK1 mediated phosphorylation of superoxide dismutase 2 (SOD2) and sirtuin 3 (SIRT3) in the presence of palmitate in mouse hepatocytes, thereby contributing to the maintenance of mitochondrial redox homeostasis.^21^ However, despite the available evidence, the molecular determinants of CDK1/CYCLIN B1 activity that coordinate mitochondrial function with cell cycle progression have not yet been elucidated, particularly in the pathogenesis of MASLD.

In the present study, we aimed to identify and characterize the functions of lncRNAs linked to the progression of MASLD. We identified mouse *Wee1-AS* and human *LNC106435.1*, which presented elevated expression levels in the livers of a murine model and of human patients with MASLD, respectively. Our findings revealed that these lncRNAs play a role in enhancing mitochondrial function by modulating cell cycle regulators such as WEE1 and CDK1/CYCLIN B1, which switch on oxidative fatty acid metabolism in the liver during the progression of MASLD.

## Results

### Identification of *Wee1-AS* that overlaps the promoter and exon 1 of the *Wee1* gene

**Fig. 1:**
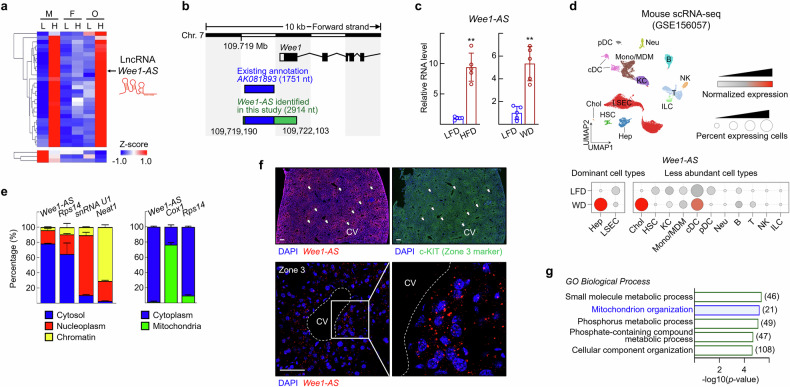
Identification of *Wee1-AS* which is overexpressed in livers of HFD-fed mice. **a** Heatmap representing 31 differentially expressed lncRNAs under HFD conditions in male and ovariectomized female mice. Each row corresponds to a single lncRNA, whereas each column represents the mean lncRNA expression of five mice from each experimental group. The scale bar denotes Z-score values, indicating relative lncRNA expression levels. Notably, *Wee1-AS* expression increased under HFD conditions, as indicated by the arrow. M: Male, F: Female, O: Ovariectomy, L: LFD, H: HFD. **b** Transcript of *Wee1-AS* was annotated previously in the NCBI database (*AK081893*). *Wee1-AS* transcripts with extra sequences were identified via rapid amplification of cDNA ends (RACE) in this study. The sizes of transcripts are shown in parentheses. The Coding Potential Calculator (https://cpc.gao-lab.org/programs/run_cpc.jsp) generated a negative coding potential score (–0.75), suggesting that the *Wee1-AS* does not encode a protein. **c** Relative *Wee1-AS* expression levels in livers of mice fed a HFD or a western diet (WD). Total RNA was isolated from the liver tissues and the levels of *Wee1-AS* were measured via qRT‒PCR. The RNA expression was normalized to that of 18srRNA. The values are presented as the means ± SDs (*n* = 5). The data were analyzed via the Mann‒Whitney test. ^**^P < 0.01. **d** Single-cell RNA sequencing (scRNA-seq) analysis of liver samples from WD-induced MASLD mice revealed 13 distinct liver cell types, including Hep (hepatocytes), Chol (cholangiocytes), HSC (hepatic stellate cells), Endo (endothelial cells), KC (Kupffer cells), Mono/MDM (monocytes and monocyte-derived macrophages), cDC (conventional dendritic cells), pDC (plasmacytoid dendritic cells), Neu (neutrophils), and ILC (innate lymphoid cells). Normalized expression levels of *Wee1-AS* across different liver cell types. Redder colors indicate higher average expression within a given cell type, whereas larger dot sizes represent a greater proportion of cells expressing *Wee1-AS*. **e** Cytosol- (blue), nucleoplasm- (red), chromatin- (yellow), cytoplasm- (dark blue), and mitochondria- (green) RNAs in subcellular fractions of primary hepatocytes were purified. The RNA levels of *Wee1-AS*, *Rps14*, *snRNA U1,* and *Neat1* was analyzed via qRT‒PCR. The proper cellular fractionation was confirmed by the localization of *Rps14*, *snRNA U1*, and *Neat1*. The data are presented as the means ± SDs (*n* = 4). **f** Localization of *Wee1-AS* in liver tissue obtained from mice in the dark cycle was analyzed via FISH. The upper images show the expression patterns of *Wee1-AS* (red) and c-Kit (green), a unique marker for the pericentral region (white arrow). The lower images provide an enhanced view, emphasizing intense clusters of *Wee1-AS* signals (red) within cytosol of individual hepatocytes surrounding the central vein (CV). Scale bar, 200 μm. **g** Gene Ontology (GO) biological process terms related to genes positively correlated with hepatic *Wee1-AS* expression in LFD- or HFD-fed mice. The bar graph illustrates significant associations between *Wee1-AS* and various biological processes. Each bar represents the -log10(*p* value), and the gene number is indicated in parenthesis

To better understand the underlying pathophysiology associated with the development of MASLD at the molecular level, we performed a global transcriptomics study of the livers of high-fat diet (HFD)-induced MASLD mice. Since the susceptibility to the development and progression of MASLD differs between men and women, we employed both male and female mice. Ovariectomized female mice were also used to assess sex-dependent susceptibility to MASLD. Among the lncRNAs, 28 were upregulated, and 3 were downregulated by HFD feeding (Fig. 1a). The detailed list of differentially expressed lncRNAs is presented in Supplementary Table 1. The lncRNAs for further study were selected on the basis of both hepatic expression levels and fold-changes in response to HFD feeding. Among the five most abundant lncRNAs, *Wee1-AS* presented the most significant expression changes in response to HFD. *Wee1-AS* is transcribed from the antisense strand of the *Wee1* locus. We characterized the entity of *Wee1-AS* via rapid amplification of cDNA ends (RACE). We identified a single-stranded transcript, which has a longer 3′- and 5′- extension than the transcript annotated in the NCBI database, *AK081893* (chromosome 7:109,719,190-109,722,103) (Fig. 1b). The expression of the lncRNA was further confirmed by qRT‒PCR, which revealed an increased level in the livers of HFD-fed or western diet-fed mice compared with LFD-fed mice (Fig. 1c).^22,23^ To explore the cell-type specificity of *Wee1-AS*, we analyzed single-cell transcriptomic data obtained from a WD-induced MASLD mouse model (GSE156057), which included 13 key liver cell types. Compared with those in LFD-fed control mice, hepatocytes presented the highest expression of *Wee1-AS*, with a notable increase in 24-week-old WD-fed mice. Among the less abundant hepatic cell types, *Wee1-AS* was also expressed in cholangiocytes and conventional dendritic cells (Fig. 1d). Similar to the scRNA-seq results, the expression of *Wee1-AS* was greater in hepatocytes and liver sinusoidal endothelial cells than in Kupffer cells or hepatic stellate cells (Supplementary Fig. 1). Considering that hepatocytes constitute approximately 80% of the liver cell population, we continued investigating *Wee1-AS* in hepatocytes. Approximately 80% of *Wee1-AS* resides in the cytoplasm and the remaining *Wee1-AS* resides in the nucleus of hepatocytes (Fig. 1e). The half-life of *Wee1-AS* in primary hepatocytes was estimated to be 0.92 h (Supplementary Fig. 2a). RNA-fluorescence in situ hybridization (FISH) analysis revealed that *Wee1-AS* was predominantly present in the region around the central vein (Fig. 1f). Compared with that in LFD-fed mice, the FISH signal in liver sections from HFD-fed mice was greater, which was consistent with the RNA level shown in Fig. 1c (Supplementary Fig. 2b). Ontology analysis of the genes whose expression level correlated with that of *Wee1-AS* revealed that the significant biological terms for the change included “mitochondrion organization” (Fig. 1g). Together, these results suggest that hepatic *Wee1-AS* may function in the control of mitochondrial energy metabolic pathways in hepatocytes located in the region near the central vein.

### *Wee1-AS* suppresses lipid accumulation and enhances mitochondrial function in the liver

**Fig. 2:**
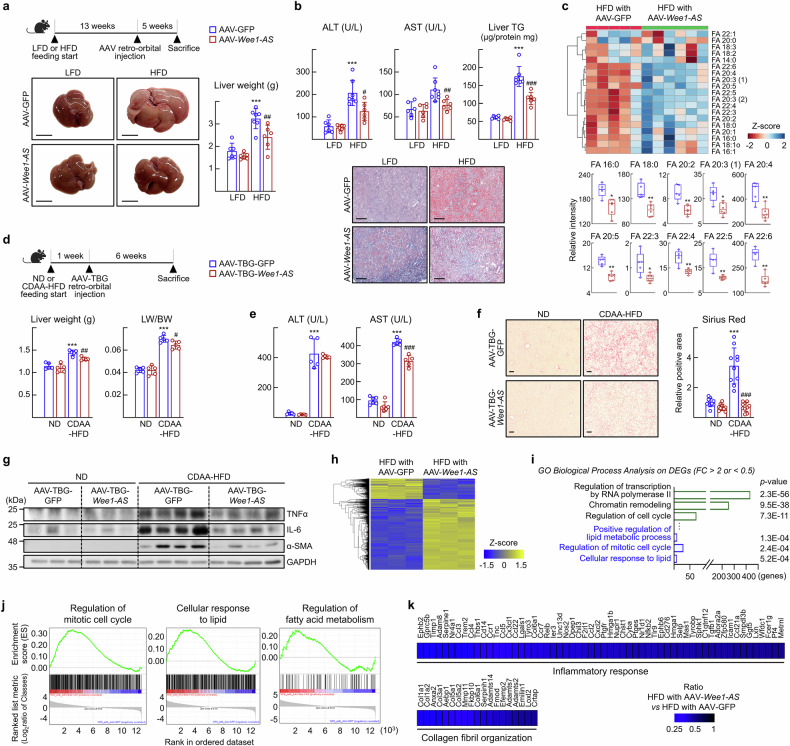
LncRNA *Wee1-AS* improves symptoms of MASLD in the HFD-fed mice. **a–c** Seven-week-old C57BL/6 N mice were fed either LFD or HFD for 18 weeks. At 13 weeks of diet feeding, AAV-*Wee1-AS* was transfused retro-orbitally. The values represent the means ± SDs (*n* = 6–7). The data were analyzed by two-way ANOVA test unless otherwise mentioned. **a** Representative images of livers and liver weights at the end of the experiments are shown. Scale bar, 1 cm. ^***^P < 0.001 vs LFD with AAV-GFP; ^##^*P* < 0.01 vs HFD with AAV-GFP. **b** Serum ALT and AST activities. ^***^P < 0.001 *vs* LFD with AAV-GFP; ^#^*P* < 0.05 and ^##^*P* < 0.01 *vs* HFD with AAV-GFP. Oil red O staining of liver sections and hepatic TG levels. Scale bar, 500 μm. ^***^P < 0.001 vs LFD with AAV-GFP; ^###^*P* < 0.001 *vs* HFD with AAV-GFP. **c** Heatmap analysis of free fatty acids in the serum. The values represent the means ± SDs (*n* = 5–6). The data were analyzed via the Mann‒Whitney test. The intensities were normalized by the median and are presented as the means ± SDs. ^*^P < 0.05 and ^**^P < 0.01. **d–g** Seven-week-old C57BL/6 N mice were fed either ND or the CDAA-HFD for 7 weeks. At 1 week of diet feeding, either AAV-TBG-GFP or AAV-TBG-*Wee1-AS* was transfused retro-orbitally. The values are represented the means ± SDs (*n* = 5). The data were analyzed via two-way ANOVA unless otherwise mentioned. **d** Liver weights and liver weight/body weight (LW/BW) ratios at the end of the experiments. ^***^P < 0.001 vs ND with AAV-TBG-GFP; ^#^*P* < 0.05 and ^##^*P* < 0.01 *vs* CDAA-HFD with AAV-TBG-GFP. **e** Serum ALT and AST activities. ^***^P < 0.001 *vs* ND with AAV-TBG-GFP; ^###^*P* < 0.001 *vs* CDAA-HFD with AAV-TBG-GFP. **f** Representative Sirius Red staining of liver sections from CDAA-HFD-fed mice infused with AAV-TBG-GFP or AAV-TBG-*Wee1-AS*. Scale bar, 100 μm. The Sirius Red-positive area was quantified in 2 images of liver tissue using ImageJ. The data were analyzed via the Mann‒Whitney test. ^***^P < 0.001 vs ND with AAV-TBG-GFP; ^###^*P* < 0.001 *vs* CDAA-HFD with AAV-TBG-GFP. **g** The expression of TNFα, IL-6, and α-SMA in liver tissues was analyzed by western blotting. **h** Liver tissues from the HFD-fed groups were subjected to RNA sequencing analysis. Clustered heatmap view of gene expression showing DEGs (FC > 2 or < 0.5, *P* < 0.05). **i** GO biological process analysis of the 4,109 differentially regulated genes (FC > 2 or < 0.5, *P* < 0.05). Bar graph showing the enriched biological processes based on the *p* value. **j** GSEA enrichment plots of representative gene sets positively correlated with the HFD with AAV- *Wee1-AS* group. **k** Transcript levels of inflammation- and fibrosis-associated genes analyzed by using RNA-sequencing in the HFD-fed group shown in Fig. 2a

To examine the function of *Wee1-AS* in the liver, adeno-associated viruses encoding *Wee1-AS* (AAV-*Wee1-AS*) were infused into HFD-fed mice. Five weeks after viral transduction, signs of MASLD improved dramatically as the liver weight, ALT and AST levels, and hepatic lipid contents decreased in the HFD-treated mice, whereas the body weight remained unchanged (Fig. 2a, b; Supplementary Fig. 3a, b). In addition, we found a significant reduction in the levels of long-chain fatty acids such as FA 16:0, FA 18:0, FA 20:2, FA 20:3 (1), FA 20:4, FA 20:5, FA 22:3, FA 22:4, FA 22:5, and FA 22:6 in the serum of the AAV-*Wee1-AS* infused mice, which suggested that the fatty acid pathway was involved in the action of *Wee1-AS* (Fig. 2c). To further investigate the effect of *Wee1-AS* on MASH progression, we conducted additional in vivo experiments in mice fed a choline-deficient, L-amino acid-defined high-fat diet (CDAA-HFD), a regimen that induces early-onset steatosis and significant liver fibrosis over a 7-week period.^24^ The infusion of AAV, whose expression was specific to hepatocytes (AAV-TBG-*Wee1-AS*), into CDAA-HFD-fed mice markedly alleviated MASH symptoms, as demonstrated by reductions in liver weight, serum AST levels, and collagen deposition, whereas body weight remained unchanged (Fig. 2d-f; Supplementary Fig. 3c, d). Furthermore, the hepatic protein levels of TNFα and IL-6 and the profibrogenic marker α-SMA were significantly decreased following viral infusion (Fig. 2g; Supplementary Fig. 3e).

Next, we analyzed global gene expression pattern changes after transfusion of AAV-*Wee1-AS* via RNA-sequencing analysis. A strong differential gene expression pattern was observed between the experimental groups (Fig. 2h). The GO biological processes of the 4427 up- or downregulated genes in the livers of the *Wee1-AS*-overexpressing group (FC > 2 or < 0.5, *P* < 0.05) were significantly associated with the biological terms “Positive regulation of lipid metabolic process,” “Regulation of mitotic cell cycle,” and “Cellular response to lipid” (Fig. 2i). The expression of genes associated with those GO terms was significantly increased, suggesting that *Wee1-AS*-induced gene expression may link lipid metabolism to the cell cycle during the progression of MASLD (Fig. 2j). Furthermore, the expression levels of genes involved in the “inflammatory response” and “collagen fibril organization” were altered upon AAV-*Wee1-AS* infusion (Fig. 2k).

**Fig. 3:**
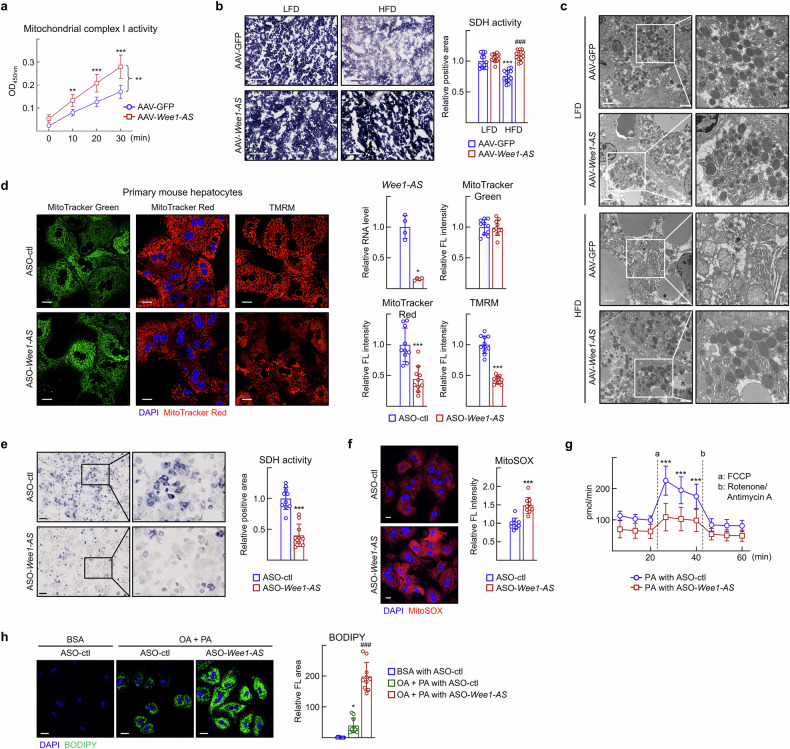
*Wee1-AS* enhances mitochondrial function in hepatocytes. **a** Activities of mitochondrial complex I in liver tissues from the mice shown in Fig. 2a. The values are presented as the means ± SDs. The data were analyzed via two-way ANOVA. ^**^P < 0.01 and ^***^P < 0.001 vs HFD with AAV-GFP. **b** Representative images of SDH activity in liver sections. Scale bar, 200 μm. The intensity was quantified in 2 images of liver tissue via ImageJ. The data were analyzed via two-way ANOVA. ^***^P < 0.001 *vs* LFD with AAV-GFP. ^###^*P* < 0.001 vs HFD with AAV-GFP. **c** Representative electron microscopy images of liver sections. Scale bar, 2 μm. **d–h** Primary hepatocytes obtained from C57BL/6 N mice were transfected with ASO-control (ctl) or ASO-*Wee1-AS*. The data were analyzed via the Mann‒Whitney test unless otherwise mentioned. **d** Cells were stained with 100 nM MitoTracker Green, MitoTracker Red CMXRos, or TMRM, and examined via a confocal microscope. Scale bar, 20 μm. The fluorescence intensity of at least 100 cells was quantified via ImageJ software. ^*^P < 0.05 and ^***^P < 0.001. **e** Representative images of SDH activity staining. Scale bar, 200 μm. The SDH-positive area in at least 300 cells was quantified via ImageJ. ^***^P < 0.001. **f** Cells were stained with 5 μM MitoSOX and analyzed via confocal microscopy. Scale bar, 20 μm. The fluorescence intensity was quantified in at least 400 cells via ImageJ. ^***^P < 0.001. **g** The oxygen consumption rate (OCR) was analyzed in the presence of palmitic acid (10 μM). The values are presented as the means ± SDs (*n* = 10). The data were analyzed by two-way ANOVA. ^***^P < 0.001 vs ASO-control (ctl). **h** Cells were treated with 0.05% BSA or free fatty acids (OA + PA, 100 μM oleic acid/50 μM palmitic acid) for 24 h. Lipid accumulation was assessed via BODIPY staining. Scale bar, 20 μm. The relative fluorescence area in at least 100 cells was quantified via Image J software. The values are presented as the means ± SDs (*n* = 10). The data were analyzed via one-way ANOVA. ^*^P < 0.05 vs BSA with ASO-control (ctl); ^###^*P* < 0.001 vs OA + PA with ASO-control (ctl)

As the decrease in lipid content was potentially due to increased lipid catabolism, we analyzed the oxidative function of the mitochondria. As expected, the activities of mitochondrial electron transport chain complex I and succinate dehydrogenase (SDH), an integral part of complex II, increased in the AAV-*Wee1-AS*-infused liver in the HFD-fed group (Fig. 3a, b). Interestingly, *Wee1-AS* infusion led to a slight increase in complex I activity in the LFD-fed group, suggesting that mitochondrial function may be modulated prior to the onset of the MASLD phenotype (Supplementary Fig. 3f). The mitochondria were swollen and the crista structure was disrupted in the hepatocytes of the HFD-fed mice, but shaped mitochondria were not observed in the AAV-*Wee1-AS*-transduced liver (Fig. 3c).

To further analyze mitochondrial function in detail, *Wee1-AS* was knocked down in primary hepatocytes. MitoTracker Green is a fluorescent probe that is commonly used to assess mitochondrial mass, whereas MitoTracker Red CMXRos is a red-fluorescent dye that stains mitochondria in live cells as its accumulation is dependent upon the membrane potential. Tetramethylrhodamine, methyl ester (TMRM) is a fluorescent dye that indicates the mitochondrial membrane potential and function. Knockdown of *Wee1-AS* with antisense oligonucleotide (ASO) in primary hepatocytes decreased the intensity of MitoTracker Red and TMRM staining, whereas it did not affect that of MitoTracker Green (Fig. 3d). These data indicated that the overall mitochondrial mass (indicated by MitoTracker Green) remained unchanged, whereas mitochondrial function (indicated by MitoTracker Red and TMRM) was reduced in the ASO-*Wee1-AS* treated group (Fig. 3d). Consistently, *Wee1-AS* knockdown suppressed SDH activity, whereas it increased MitoSOX staining, an indicator of mitochondrial ROS, in primary hepatocytes (Fig. 3e, f). Next, we assessed the oxygen consumption rate (OCR) of hepatocytes treated with palmitic acid. While the basal OCR did not significantly differ, the uncoupled OCR was markedly reduced in the ASO*-Wee1-AS* transfected hepatocytes, suggesting a critical role of *Wee1-AS* in oxidative metabolism (Fig. 3g). Finally, we measured the amount of intracellular lipids. As shown in Fig. 3h, fatty acid treatment induced lipid accumulation in hepatocytes, which was further enhanced after ASO-*Wee1-AS* transfection (Fig. 3h). Together, these results indicate that *Wee1-AS* improves mitochondrial function and oxidative metabolism and thereby suppresses hepatic lipid accumulation.

### *Wee1-AS* regulates CDK1 activity as well as the protein stability of CYCLIN B1

**Fig. 4:**
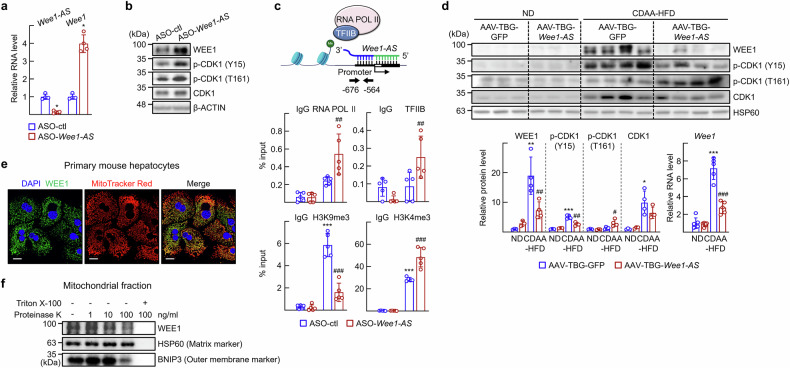
Knockdown of *Wee1-AS* causes increases in WEE1 levels and inactivation of CDK1. **a–c** Primary hepatocytes were transfected with ASO-control (ctl) or ASO-*Wee1-AS*. The values are presented as the means ± SDs (*n* = 4). The data were analyzed via the Mann‒Whitney test unless otherwise mentioned. **a** The *Wee1* mRNA level was measured via qRT‒PCR. ^*^P < 0.05 vs ASO-control (ctl). **b** Expression of the indicated proteins was analyzed by western blotting. **c** DNA fragments encoding the *Wee1* gene promoter were immunoprecipitated with the indicated antibodies and then amplified via qPCR with specific primers. The data were analyzed via two-way ANOVA. ^***^P < 0.001 vs IgG of the ASO-control (ctl). ^##^*P* < 0.01 and ^###^*P* < 0.001 *vs* anti-RNA pol II, anti-TFIIB, anti-H3K9me3, or anti-H3K4me3 of the ASO-control. **d** Tissue extracts were prepared from livers of the mice shown in Fig. 2d. The expression of the indicated proteins was analyzed by western blotting. The *Wee1* mRNA level was measured by qRT‒PCR. The data were analyzed via two-way ANOVA. ^*^P < 0.05, ^**^P < 0.01, and ^***^P < 0.001 vs ND with AAV-TBG-GFP; ^#^*P* < 0.05, ^##^*P* < 0.01, and ^###^*P* < 0.001 vs CDAA-HFD with AAV-TBG-GFP. **e** Primary hepatocytes obtained from C57BL/6 N mice were stained with 100 nM MitoTracker Red CMXRos and WEE1 immunostaining was performed (green). Scale bar, 20 μm. **f** Mitochondria of primary hepatocytes obtained from C57BL/6 N mice were digested with proteinase K (1 ng/ml, 10 ng/ml, and 100 ng/ml) with or without 10% Triton X-100 for 1 h at RT. The samples were subsequently analyzed via western blotting with the indicated antibodies. The purity of the mitochondrial fractions was validated by evaluating the expression of specific mitochondrial marker proteins as shown in Supplementary Fig. 4b

As *Wee1-AS* overlays the promoter region of the *Wee1* gene, we asked whether *Wee1-AS* regulated the transcription of *Wee1*. When *Wee1-AS* knocked down with ASO, the level of *Wee1* mRNA increased in primary hepatocytes (Fig. 4a). The protein level of WEE1 as well as the subsequent phosphorylation of CDK1 (Y15) increased in the ASO-*Wee1-AS* treated hepatocytes (Fig. 4b; Supplementary Fig. 4a). Next, to understand the mechanism by which *Wee1-AS* suppresses *Wee1* transcription, we examined the recruitment of the basal transcription machinery to the *Wee1* promoter region. Chromatin immunoprecipitation (ChIP) assays revealed that the recruitment of RNA polymerase II and TFIIB, components of the basal transcription machinery, was increased when *Wee1-AS* was knocked down, suggesting that the complementary binding between *Wee1-AS* and the promoter of *Wee1* interfered with the recruitment of the basal transcription machinery. The activation of *Wee1* transcription in the absence of *Wee1-AS* was further demonstrated by epigenetic changes such as decreased H3K9me3 but increased H3K4me3 levels in the *Wee1* promoter. Together, these results suggested that *Wee1-AS* suppressed the transcription of *Wee1* by interfering binding of the basal transcription machinery to the promoter of the *Wee1* gene (Fig. 4c). Interestingly, both the protein and the mRNA levels of Wee1 increased in the livers of the CDAA-HFD-fed mice (Fig. 4d). The prominent induction of WEE1 protein in MASH conditions suggested a potential role of these proteins in the pathogenesis of diet-induced MASLD. The level of p-CDK1 (Y15) decreased, but the level of active p-CDK1 (T161) increased after AAV-TBG-*Wee1-AS* transduction in the CDAA-HFD-fed mice (Fig. 4d). Next, we examined subcellular compartment in which WEE1 resides. Strikingly, we found that a substantial portion of WEE1 colocalized with mitochondria (Fig. 4e; Supplementary Fig. 4b). Additionally, a proteinase K digestion assay suggested that WEE1 resides in the outer membrane of mitochondria (Fig. 4f). To elucidate the causal role of Wee1 in lipid metabolism and mitochondrial function, we performed an in vivo *Wee1* knockdown experiment by transducing AAV-TBG-shWee1 into CDAA-HFD-fed mice. Six weeks after viral transduction, the serum ALT levels and hepatic lipid contents were markedly reduced in the CDAA-HFD-fed mice. Moreover, the activities of SDH and the expression levels of electron chain transport components, such as ATP synthase, H+ transporting, mitochondrial F1 complex, alpha 1 (ATP5A) and mitochondrially encoded cytochrome c oxidase I (MTCO1) were significantly increased following *Wee1* knockdown (Supplementary Fig. 5a–g). Together, these findings indicate that Wee1 contributes to the regulation of hepatic lipid metabolism and mitochondrial function. Mechanistically, *Wee1* knockdown reduced the level of p-CDK1 (Y15) while increasing the level of p-CDK1 (T161) (Supplementary Fig. 5h). Moreover, WEE1 overexpression in primary hepatocytes increased p-CDK1 (Y15) levels, supporting the link between mitochondrial WEE1 and its regulatory role in CDK1 activity (Supplementary Fig. 5i).

**Fig. 5:**
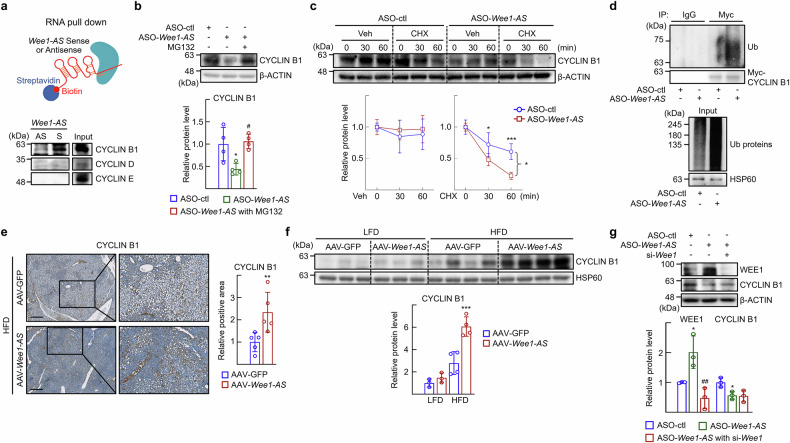
*Wee1-AS* increases the stability of CYCLIN B1 and its translocation to mitochondria. **a** RNA pull-down assays were carried out with biotinylated *Wee1-AS* (Sense, S) or its antisense (AS) with protein lysates prepared from mouse liver tissues. Proteins pulled down with the biotinylated *Wee1-AS* or its antisense control were analyzed by western blotting (bottom). **b**, **c** Primary hepatocytes were transfected with ASO-control (ctl) or ASO-*Wee1-AS*. **b** Cells were treated with DMSO (Veh) or 20 μM MG132 for 3 h. The values are represented the means ± SDs (*n* = 4). The expression of CYCLIN B1 was analyzed via western blotting. The data were analyzed by one-way ANOVA. ^*^P < 0.05 vs ASO-control (ctl); ^#^*P* < 0.05 *vs* ASO-*Wee1-AS*. **c** Cells were treated with ethanol (Veh) or 50 μM cycloheximide (CHX) for the indicated times. The expression of the indicated proteins was analyzed via western blotting. The data were analyzed by two-way ANOVA. ^*^P < 0.05 and ^***^P < 0.001 *vs* ASO-control (ctl). **d** AML12 cells were transfected with Myc-CYCLIN B1 and ASO-*Wee1-AS* and treated with 10 μM MG132 for 3 h. Immunoprecipitation (IP) was performed with an IgG or anti-Myc antibody and probed with an anti-ubiquitin antibody. **e** Representative images of immunohistochemical staining of CYCLIN B1 in liver sections from the mice shown in Fig. 2a. Scale bar, 500 μm. The intensity of CYCLIN B1 staining was quantified in images from 5 mice via ImageJ. The data were analyzed via the Mann‒Whitney test. ^**^P < 0.01 *vs* HFD with AAV-GFP. **f** Tissue extracts were prepared from the liver of the mice shown in Fig. 2a. The expression of CYCLIN B1 was analyzed via western blotting. The values are represented the means ± SDs (*n* = 3–4). The data were analyzed via two-way ANOVA. ^***^P < 0.001 vs HFD with AAV-GFP. **g** Primary hepatocytes were transfected with ASO-*Wee1-AS* and si-*Wee1*. The expression of the indicated proteins was analyzed via western blotting. The data were analyzed by one-way ANOVA. ^*^P < 0.05 vs ASO-control (ctl) with si-control and ^##^*P* < 0.01 vs ASO-*Wee1-AS* with si-control

Given that *Wee1-AS* knockdown resulted in a reduction in CDK1 activity, we asked whether cyclins were also involved in the function of *Wee1-AS*. Thus, we performed RNA pull-down assays with in vitro transcribed *Wee1-AS*. As shown in Fig. 5a, *Wee1-AS* interacted with CYCLIN B1, a key protein responsible for the activation of CDK1 (Fig. 5a). We also observed that the protein level of CYCLIN B1 decreased in the presence of ASO-*Wee1-AS*, but it was restored in the presence of MG132 (Fig. 5b). When protein synthesis was blocked by cycloheximide, the degradation of the CYCLIN B1 protein was faster in the presence of ASO-*Wee1-AS*, and the ubiquitination of CYCLIN B1 was markedly increased (Fig. 5c, d). Together, these results indicated that *Wee1-AS* bound to CYCLIN B1, which suppressed the ubiquitin/proteasome pathway-mediated degradation of CYCLIN B1. Consistently, the expression of CYCLIN B1 was largely increased in the livers of the AAV-*Wee1-AS*-transduced mice (Fig. 5e, f). *Wee1-AS*-mediated CYCLIN B1 stability was independent of the function of WEE1, as the knockdown of *Wee1* did not affect the CYCLIN B1 expression level (Fig. 5g).

### Mitochondrial CDK1/CYCLIN B1 is activated in the presence of *Wee1-AS*

**Fig. 6:**
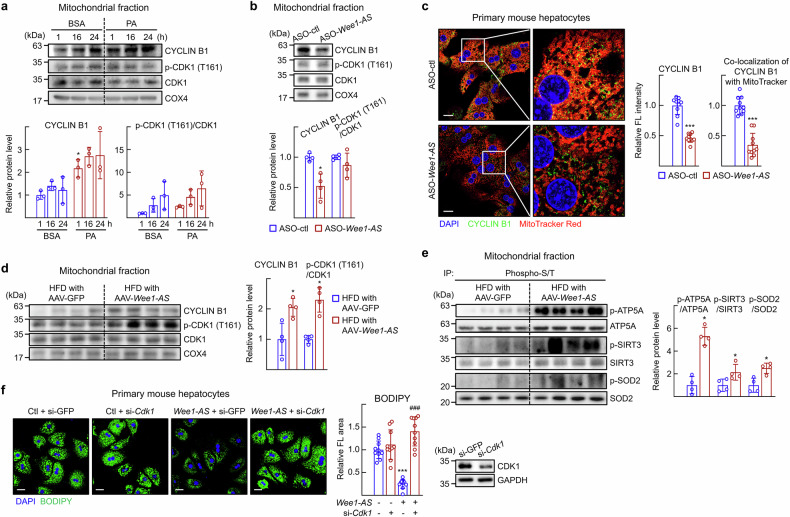
*Wee1-AS* enhances mitochondrial homeostasis via activation of CDK1/CYCLIN B1. **a** Primary hepatocytes were treated with 25 μM palmitic acid (PA)-conjugated with 0.05% bovine serum albumin (BSA) for the indicated times. The mitochondrial fraction was prepared and analyzed via western blotting. The values are represented the means ± SDs (*n* = 3). The data were analyzed via two-way ANOVA. ^*^P < 0.05 *vs* BSA treatment for 1 h. **b**, **c** Primary hepatocytes were transfected with ASO-control (ctl) or ASO-*Wee1-AS*. The values are presented as the means ± SDs (*n* = 4). The data were analyzed via the Mann‒Whitney test. **b** The mitochondrial fraction was prepared and analyzed by western blotting. The intensity of each protein band was quantified via ImageJ and normalized to that of COX4 or CDK1. ^*^P < 0.05 vs ASO-control (ctl). **c** Cells were stained with 100 nM MitoTracker Red CMXRos (red). Immunostaining was performed for CYCLIN B1 (green). Scale bar, 20 μm. The fluorescence intensity in at least 100 cells was quantified via Image J software. The percentage of co-localization was determined via the JACoP plugin of ImageJ software. The amount of mitochondrial CYCLIN B1 was normalized to the intensity of MitoTracker signal. ^***^P < 0.001 vs ASO-control (ctl). **d**, **e** Tissue extracts were prepared from the livers of the mice shown in Fig. 2a. The values are presented as the means ± SDs (*n* = 4). The data were analyzed via the Mann‒Whitney test. **d** The mitochondrial fraction was prepared and analyzed via western blotting. ^*^P < 0.05 vs HFD with AAV-GFP. **e** The mitochondrial fraction was prepared and the phosphorylation of the indicated proteins was analyzed via immunoprecipitation (IP) with an anti-phospho-serine/threonine antibody and probed with specific antibodies. The lanes were run on the same gel but were noncontiguous. The intensity of each protein band was quantified via ImageJ. ^*^P < 0.05 *vs* HFD with AAV-GFP. **f** Primary hepatocytes were transfected with si-GFP/si-*Cdk1* or pcDNA-empty vector (Ctl)/pcDNA-*Wee1-AS* (*Wee1-AS*), and then treated with free fatty acids (200 μM oleic acid/100 μM palmitic acid) for 24 h. Lipid accumulation was assessed via BODIPY staining. Scale bar, 20 μm. The relative fluorescence area in at least 100 cells was quantified via Image J software. The values are presented as the means ± SDs (*n* = 10). The data were analyzed via two-way ANOVA. ^***^P < 0.001 vs pcDNA-empty vector (Ctl) with si-GFP; ^###^*P* < 0.001 vs pcDNA-*Wee1-AS* (*Wee1-AS*) with si-GFP

Previously, it was reported that CDK1/CYCLIN B1 is translocated into mitochondria and enhances mitochondrial function by phosphorylating key elements to maintain oxidative stress.^20,25^ Therefore, we asked whether the function of *Wee1-AS* was associated with the mitochondrial translocation of CDK1/CYCLIN B1. First, when mitochondria were fractionated from primary hepatocytes, an increase in mitochondrial CYCLIN B1 was clearly observed, particularly in the presence of palmitic acid (Fig. 6a). The amount of mitochondrial CYCLIN B1 decreased in the ASO-*Wee1-AS*-treated hepatocytes (Fig. 6b). Consistently, immunofluorescence studies revealed that the amount of CYCLIN B1 that colocalized with MitoTracker was reduced in the absence of *Wee1-AS*, demonstrating that *Wee1-AS* increased the level of mitochondrial CYCLIN B1 (Fig. 6c). Similarly, increases in the levels of mitochondrial CYCLIN B1 and p-CDK1 (T161) were observed in livers of *Wee1-AS* overexpressing mice (Fig. 6d). The phosphorylation of CDK1 downstream substrates such as p-ATP5A, p-SIRT3, and p-SOD2, which indicate increased function and lower oxidative stress in the mitochondria, was largely increased in the AAV-*Wee1-AS-*transfused liver in vivo (Fig. 6e).^21,25^ Finally, *Wee1-AS* overexpression decreased lipid accumulation, whereas Cdk1 silencing attenuated the reduction in lipid accumulation caused by *Wee1-AS* overexpression (Fig. 6f). These data suggested that the reduction in lipids caused by *Wee1-*AS was associated with the CDK1/CYCLIN B1 pathway in hepatocytes.

### Adavosertib, a WEE1 inhibitor, improves mitochondrial function and attenuates the signs of MASLD in mice

**Fig. 7:**
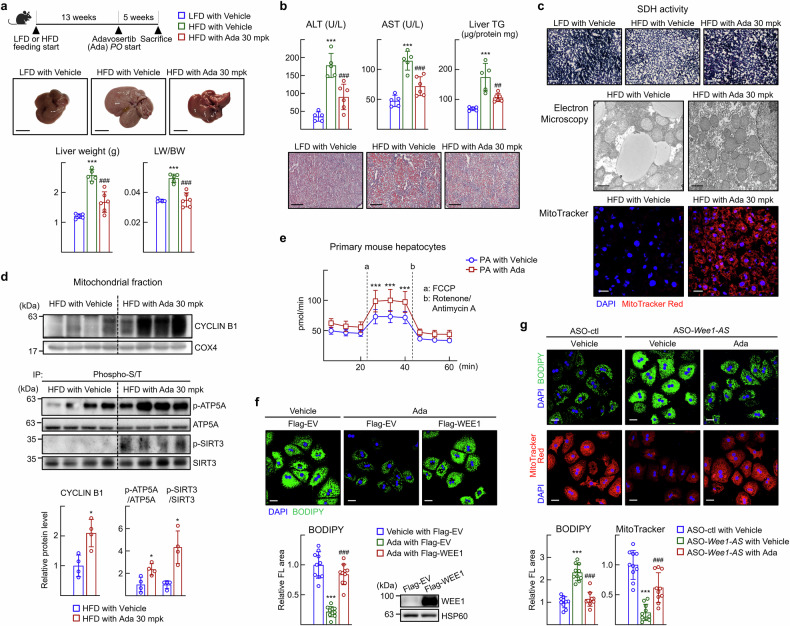
Adavosertib improves mitochondrial function and attenuates HFD-induced MASLD in mice. **a–d** Seven-week-old C57BL/6 N mice were fed either LFD or HFD for 18 weeks. At 13 weeks of diet feeding, adavosertib (30 mg/kg body weight) or vehicle (0.5% methylcellulose) was orally administered daily for 5 weeks. This dose of adavosertib did not significantly change in the serum AST or ALT levels in LFD-fed mice. The values are presented as the means ± SDs (*n* = 5–6). The data were analyzed via one-way ANOVA unless otherwise mentioned. **a** Representative images of the livers, liver weights, and liver weight/body weight (LW/BW) ratios of the experimental mice are shown. Scale bar, 1 cm. ^***^P < 0.001 *vs* LFD with Vehicle; ^###^*P* < 0.001 *vs* HFD with Vehicle. **b** Representative images of Oil red O stained liver sections and hepatic TG levels. Scale bar, 500 μm. Serum ALT and AST activities were measured. ^***^P < 0.001 vs LFD with Vehicle; ^##^*P* < 0.01 and ^###^*P* < 0.001 *vs* HFD with Vehicle. **c** Representative images of SDH staining in liver sections. Scale bar, 100 μm. Representative electron microscopy images of liver sections. Scale bar, 1 μm. Liver tissues were stained with 10 nM MitoTracker Red CMXRos (red) and DAPI (blue). Scale bar, 20 μm. **d** Tissue extracts were prepared from the livers of HFD-fed mice. The mitochondrial fraction was prepared and analyzed via western blotting. The data were analyzed via the Mann‒Whitney test. ^*^P < 0.05 vs HFD with Vehicle. Phosphorylation of the indicated proteins in the mitochondrial fraction was analyzed by immunoprecipitation (IP) using an anti-phospho-serine/threonine antibody and probed with specific antibodies. The lanes were run on the same gel but were noncontiguous. Intensity of p-ATP5A/ATP5A and p-SIRT3/SIRT3 band was quantified via ImageJ. ^*^P < 0.05 vs HFD with Vehicle. **e** Primary hepatocytes were treated with adavosertib 100 nM for 24 h. The absence of DNA damage at this dose was confirmed by γH2AX assessment. The oxygen consumption rate (OCR) was analyzed in the presence of 10 μM palmitic acid. The values are represented as the means ± SDs (*n* = 6). The data were analyzed via two-way ANOVA. ^***^P < 0.001 *vs* PA with vehicle. **f** Primary hepatocytes were transfected with a Flag-empty vector (EV) or Flag-WEE1. Then, the cells were treated with free fatty acids (200 μM oleic acid/100 μM palmitic acid) and 100 nM adavosertib for 24 h. Lipid accumulation was assessed via BODIPY staining. Scale bar, 20 μm. The relative fluorescence area in at least 100 cells was quantified via Image J software. The values are represented as the means ± SDs (*n* = 10). The data were analyzed via one-way ANOVA. ^***^P < 0.001 *vs* vehicle with Flag-empty vector (EV); ^###^*P* < 0.001 vs adavosertib (Ada) with Flag-empty vector (EV). **g** Primary hepatocytes were transfected with ASO-control (ctl) or ASO-*Wee1-AS*. Then, the cells were treated with free fatty acids (100 μM oleic acid/50 μM palmitic acid) and 100 nM of adavosertib for 24 h. Lipid accumulation was assessed via BODIPY staining, and mitochondrial function was assessed via MitoTracker Red staining. Scale bar, 20 μm. The relative fluorescence area in at least 100 cells was quantified via Image J software. The values are represented as the means ± SDs (*n* = 10). The data were analyzed via one-way ANOVA. ^***^P < 0.001 vs ASO-ctl with vehicle; ^###^*P* < 0.001 vs ASO-*Wee1-AS* with vehicle

Adavosertib is the first-in-class WEE1 inhibitor that has shown antitumor efficacy with the best response in ovarian and endometrial cancers.^26^ As *Wee1-AS* led to increased mitochondrial function and improved signs of MASLD, we asked whether adavosertib had beneficial effects on MASLD in mice. Administration of adavosertib at a dose of 30 mg/kg reduced the gained liver weight by 50% in the HFD-fed mice, whereas the body weight remained unchanged (Fig. 7a; Supplementary Fig. 6a). The accumulation of neutral lipids was suppressed and the levels of ALT and AST returned to normal ranges after adavosertib treatment (Fig. 7b). The decreased SDH activity and swollen mitochondrial morphology in the hepatocytes of HFD-fed mice recovered to normal after adavosertib treatment (Fig. 7c; Supplementary Fig. 6b). In addition, MitoTracker Red staining increased after adavosertib treatment, confirming that enhanced mitochondrial function occurred through the inhibition of WEE1 activity (Fig. 7c). The levels of mitochondrial CYCLIN B1, p-ATP5A, and p-SIRT3 increased after adavosertib treatment (Fig. 7d). Next, we evaluated the effect of adavosertib on fatty acid oxidation by measuring the OCR. Treatment with adavosertib in the presence of palmitic acid significantly increased the uncoupled OCR in primary hepatocytes (Fig. 7e). Adavosertib treatment largely decreased lipid accumulation; however additional Wee1 overexpression restored the level of lipids in hepatocytes (Fig. 7f). Notably, ASO-*Wee1-AS*-induced lipid accumulation and dysregulation of mitochondria were reversed by treatment with adavosertib, indicating a functional link between *Wee1-AS* and WEE1 in the regulation of fatty acid oxidation (Fig. 7g). These data suggested that the effects of adavosertib were likely mediated by WEE1. Together, these results suggest that the inhibition of WEE1 may provide a novel strategy to enhance mitochondrial function and improve lipid metabolism in the liver.

### *LNC106435.1*, a human analog of *Wee1-AS*, enhances mitochondrial function

**Fig. 8:**
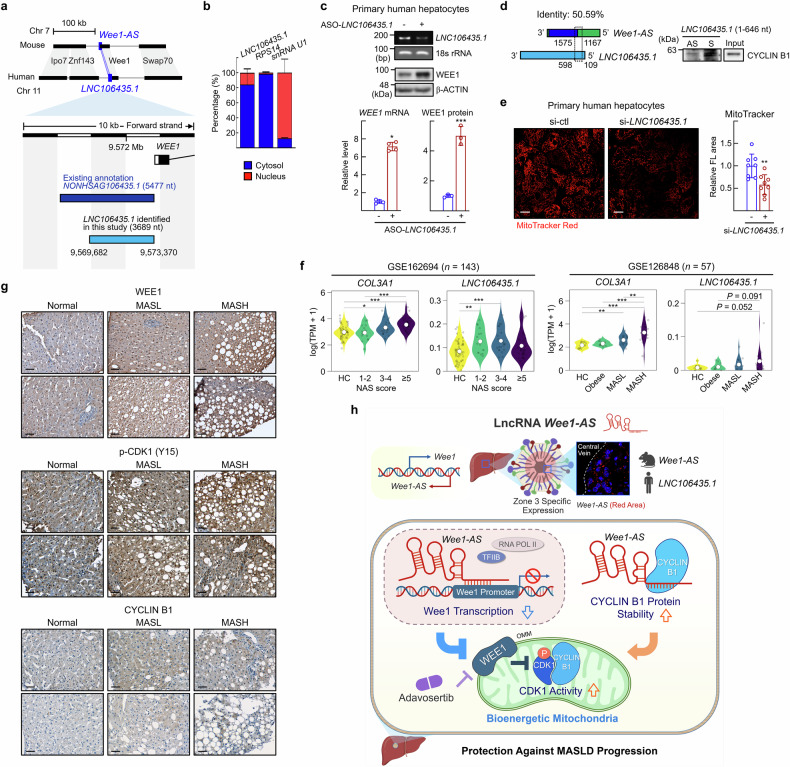
Knockdown of *LNC106435.1*, a human analog of *Wee1-AS*, suppresses mitochondrial function. **a** Transcript of *LNC106435.1* was annotated previously (NCBI Reference Sequence *NONHSAG106435.1*) (dark blue). In this study, *LNC106435.1* transcripts were identified from Huh7 cells via RACE (light blue). The size of the transcript is shown in parenthesis (bottom). **b** Cytosol- (blue) and nucleus- (red) RNAs in the subcellular fractions of primary hepatocytes were purified. The RNA levels of *LNC106435.1*, *RPS14*, and *snRNA U1* were analyzed via qRT‒PCR. The proper cellular fractionation was confirmed by localization of *RPS14* and *snRNA U1*. The data are represented as the means ± SDs (*n* = 4). **c** Primary human hepatocytes were transfected with ASO-control (ctl) or ASO-*LNC106435.1*. The RNA levels of *LNC106435.1* were measured by RT‒PCR. The RNA and protein levels of Wee1 were analyzed via qRT‒PCR and western blotting, respectively. **d** RNA pull-down assays were carried out with biotinylated *LNC106435.1* (1-646 nt, within which the sequence is conserved in mouse *Wee1-AS* with 50.59% identity) (sense, S) or its antisense (AS) with protein lysates prepared from Huh7 cells. Pull-down proteins were analyzed via western blotting. **e** Primary human hepatocytes were transfected with si-control (ctl) or si-*LNC106435.1*. The cells were stained with 100 nM MitoTracker Red CMXRos, and examined via a confocal microscope. Scale bar, 20 μm. Representative images are shown. The fluorescence intensity in at least 100 cells was quantified via Image J software. The data were analyzed via the Mann‒Whitney test. ^**^P < 0.01 *vs* si-control (ctl). **f** The expression levels of *LNC106435.1* were analyzed via two publicly available NCBI GEO datasets, namely, GSE162694, which included 143 individuals categorized as healthy controls (HCs) or patients with NAFLD activity scores (NASs) ranging from 1 to 7. Patients were further classified as mild MASLD (NAS 1–2), borderline MASLD (NAS 3–4), and advanced MASH (NAS ≥ 5), and GSE126848 which included 57 individuals comprising HCs and patients with obesity, MASL, or MASH. The transcript abundance of *COL3A1* (positive control) and *LNC106435.1* was quantified via Salmon software and expressed as log(TPM + 1). The white circles represent the mean values. The data were analyzed via the *t* test. ^*^P < 0.05, ^**^P < 0.01, and ^***^P < 0.001. **g** Representative images of immunohistochemical staining of WEE1, p-CDK1 (Y15), and CYCLIN B1 in human liver tissue samples from normal, MASL, and MASH patients. Scale bar, 50 μm. **h** Schematic illustration of how the spatiotemporal lncRNA *Wee1-AS* controls mitochondrial function through the activation of CDK1/CYCLIN B1 (created with BioRender.com)

Finally, we searched for a human analog of *Wee1-AS* that functions in hepatic lipid metabolism in humans. We employed a sequence-based approach using LNCipedia, a comprehensive database of annotated lncRNAs. This analysis identified *LNC106435.1*, originally *NONHSAG106435.1*, as the human lncRNA with the highest homology to mouse *Wee1-AS*. *LNC106435.1* overlaps with the promoter region of the human *WEE1* gene (Fig. 8a). Like *Wee1-AS*, 80% of *LNC106435.1* resided in the cytosol in human Huh7 cells (Fig. 8b). Knockdown of *LNC106435.1* with ASO increased the Wee1 levels of both RNA and protein in primary human hepatocytes (Fig. 8c). A 5’-end fragment of *LNC106435.1* (nt 109-598), which shows 50.59% homology with mouse *Wee1-AS*, interacted with CYCLIN B1 (Fig. 8d). Consistent with this finding, a *Wee1-AS* mutant lacking the conserved sequence (nt 1167-2003), failed to bind to CYCLIN B1, indicating that this region serves as the binding site for CYCLIN B1 (Supplementary Fig. 7). Knockdown of *LNC106435.1* reduced MitoTracker Red staining, indicating that *LNC106435.1* is a functional analog of *Wee1-AS* (Fig. 8e).

To assess the significance of human *LNC106435.1* in the pathophysiology of MASLD, we analyzed two public databases (GSE162694 and GSE126848) containing liver transcriptomic profiles from 200 individuals with varying MASLD activity scores. Our analysis revealed that *LNC106435.1* expression was significantly or marginally increased in both mild MASL patients and advanced MASH patients compared with healthy controls (Fig. 8f). A Jonckheere–Terpstra trend test on GSE126848 confirmed a significant monotonic increase from healthy controls to MASH patients (*P* = 0.049), supporting the association of *LNC106435.1* with disease progression.^27^ Additionally, we performed immunohistochemistry to assess WEE1/p-CDK1 (Y15)/CYCLIN B1 expression in liver samples from normal individuals and patients with hepatic steatosis and MASH obtained from the clinic (Supplementary Table 2). As shown in Fig. 8g, WEE1/p-CDK1 (Y15)/CYCLIN B1 protein levels increased progressively with MASLD severity (Fig. 8g). These findings highlight the clinical relevance of *LNC106435.1* in MASLD pathobiology.

## Discussion

Here, we identified a novel lncRNA *Wee1-AS* that coordinates oxidative fatty acid catabolism through dual modal activation of mitochondrial CDK1/CYCLIN B in hepatocytes under excess nutrient supply. This lncRNA provides a regulatory mechanism for the WEE1-CDK1/ CYCLIN B axis that may link mitochondrial function to cell cycle regulation during the progression of MASLD as summarized in Fig. 8h.

Metabolic homeostasis can be established, in part, by the spatial compartmentalization of metabolic pathways, known as liver zonation. The liver lobule can be divided into the periportal (zone 1), midlobular (zone 2), and pericentral regions (zone 3), which exhibit distinct metabolic activities that reflect the functional heterogeneity of hepatocytes along the lobular axis.^28^ During development of MASLD, steatosis and inflammation initially arise in the pericentral region.^29^ The observation that key metabolic players potentially influence on the progression of MASLD, such as peroxisome proliferator-activated receptor and farnesoid X receptor, are predominantly expressed in the pericentral region, underscores the important metabolic role of the pericentral region in the pathological context of MASLD.^29^ The *Wee1-AS* expression level was particularly high in the pericentral region of the liver, suggesting the critical role of *Wee1-AS* in maintaining mitochondrial quality control and oxidative fatty acid catabolism in this region. We observed that the hepatic expression levels of both *Wee1-AS* and its downstream Wee1 were elevated under the pathological conditions of MASLD, which may indicate the presence of a compensatory mechanism in the regulation of these genes under disease conditions. Identification of upstream factors that regulate *Wee1-AS* expression could significantly increase our understanding of nutrient-sensing-shaped zonal mechanisms in normal physiology as well as in the pathogenesis of MASLD.

WEE1 is a kinase that plays a key role in the G2/M cell cycle checkpoint, preventing mitotic entry in response to DNA damage.^30^ Here, we revealed several novel molecular aspects of WEE1 and its regulatory mechanisms. First, we identified a new lncRNA, *Wee1-AS*, that regulates the transcription of the *Wee1* gene by interfering with the formation of a transcription initiation complex through antisense positioning in the *Wee1* gene promoter. To the best of our knowledge, *Wee1-AS* is the first lncRNA that regulates *Wee1* gene expression. We also identified a human homolog of *Wee1-AS, LNC106435.1*, which is transcribed from the antisense strand of the human *Wee1* gene promoter. Second, unexpectedly, the WEE1 protein is present in the mitochondria, probably in the outer membrane. We showed that knockdown of *Wee1* enhances CDK1/CYCLIN B1 activity, which increases mitochondrial function through maintaining mitochondrial redox homeostasis. Thus, the cellular function of WEE1 could extend to the regulation of oxidative fatty acid catabolism beyond the well characterized cell cycle regulation. Third, we observed that adavosertib, a WEE1 inhibitor, enhanced mitochondrial function, probably by blocking WEE1-mediated suppression of CDK1. To date, Adavosertib has been shown to display wide antitumor efficacy among advanced solid tumors.^26^ In this study, we showed for the first time that adavosertib treatment led to the amelioration of MASLD in a mouse disease model. Together, these observations may support WEE1 as a novel target for developing therapeutic strategies to treat patients with MASLD. However, additional studies are needed to further elucidate the metabolic regulatory roles of WEE1 in depth.

The cell cycle is an intricately coordinated process that is crucial for the restoration of adequate liver function after liver injury.^31^ The liver with MASLD is characterized by lipid droplet accumulation within hepatocytes, which is accompanied by both metabolic dysregulation and disturbances in cellular division.^32^ Hepatocytes situated near the central vein display greater proliferative potential than those in the mid-lobular or periportal regions do, which is likely to compensate for the damage frequently occurring in this region.^33^ We found that WEE1 expression increased in HFD-induced MASLD livers. Interestingly, marked nuclear translocation of WEE1 was noted, particularly in hepatocytes residing in the pericentral region as shown in Supplementary Fig. 8. As WEE1 in the nucleus can suppress the CDK1 activity required for the G2/M transition, it implies impaired cell cycle progression in hepatocytes in the pericentral region. When *Wee1-AS* was transduced via the AAV delivery system, WEE1 resided mostly in the cytoplasm and the expression level of CYCLIN B1 increased at this pericentral region, suggesting that the proliferation of hepatocytes, which was halted in the MASLD liver, was reinitiated by the overexpression of *Wee1-AS*. Further studies on the shuttling of WEE1 between mitochondria and the nucleus may uncover the link between the cell cycle and metabolism.

Recently, noncoding RNAs have emerged as unique targets for therapeutic development because of their unique cell- or tissue-specific expression patterns. Moreover, sequence-based nucleic acid therapeutics are advancing rapidly. Thus, the identification and evaluation of lncRNA targets have potential for translation into clinical applications within a reasonable time frame.^34^ Here, we identified *LNC106435.1*, located on chromosome 11:9,569,682-9,573,370, as the human homolog corresponding to *Wee1-AS* in mice. Notably, we demonstrated that the knockdown of *Wee1-AS* or its human homolog, *LNC106435.1*, resulted in impaired mitochondrial function in both mouse and human hepatocytes. Specifically, the regions of mouse *Wee1-AS* and human *LNC106435.1* with 50.59% homology, interact with CYCLIN B1, indicating that the conserved sequences are essential for enhancing mitochondrial function. Thus, the development of therapeutic strategies targeting this conserved sequence appears feasible, potentially offering a novel lncRNA-based therapeutic approach for the treatment of MASLD. In addition, we found that *Wee1-AS* highly expressed in the heart and muscle in which mitochondrial function is essential for normal physiology. Since MASLD induces a vicious interorgan cross-talk between multiple metabolic organs such as muscle and heart, *Wee1-AS* could be a valuable therapeutic target for MASLD treatment. However, several questions still need to be addressed in our study. For example, increase in the level of the Ub protein after *Wee1-AS* was knocked down, as shown in Fig. 5, may suggest that autophagy is one of the mechanisms of action of *Wee1-AS*. Since ubiquitination is a prerequisite for the autophagic degradation of certain proteins and *Wee1-AS* knockdown also leads to the accumulation of the p62/SQSTM1 protein, an adaptor protein of selective autophagy, as shown in Supplementary Fig. 9, the involvement of *Wee1*-AS in autophagy should be investigated. In addition, future research should validate the association between *LNC106435.1* and MASLD in large-scale cohort studies to strengthen the clinical relevance of our findings.

## Materials and methods

### Ethics declarations

All animal experiments were approved by the Seoul National University Institutional Animal Care and Use Committee (permission numbers SNU-220211-1 and SNU-230528-1-3), and conducted according to the committee’s guidelines. The use of human tissue samples was approved by the institutional review board of the Seoul Metropolitan Government Boramae Medical Center (IRB No. 16-2014-86), and informed consent was obtained from all donors.

### Identification of differentially expressed hepatic lncRNAs in the mouse MASLD model

Wild-type male and female C57BL/6 N mice (6–8 weeks old) were purchased from Jackson Laboratories (Bar Harbor, ME, USA) and housed in a specific-pathogen-free room at 22–24 °C and 50–60% humidity with a 12 h light/dark cycle. Both a HFD (D12492) and an LFD (D12450J) (Research Diets, New Brunswick, NJ, USA) were fed to seven-week-old mice for 12 weeks. A group of female mice was ovariectomized one week before the beginning of the specific diet. At the end of the diet, the mice were sacrificed by CO_2_ euthanasia, and the livers were removed. The MASLD phenotypes of these mice were described previously.^23^ The global lncRNA transcriptomes of isolated mouse hepatic RNAs were analyzed using a Mouse V4.0 LncRNA Microarray, which can detect 37,949 lncRNAs and 22,692 mRNAs (Arraystar Inc., Rockville, MD, USA). Quantile log2 normalization for gene expression was conducted, and the fold change between each group was calculated. The expression levels of a total of 20,134 lncRNAs were normalized and converted into fold changes of HFD/low-fat diet (LFD) groups. Among the lncRNAs, 28 lncRNAs were upregulated, and 3 lncRNAs were downregulated by HFD feeding with the following criteria: *p* < 0.05 according to two-way ANOVA considering diet and sex; HFD/LFD fold change >2 for both males and ovariectomized; and HFD/LFD fold change >0.5 and <2 for females. A heatmap was drawn using the expression of 31 selected lncRNAs in each experimental group under hierarchical clustering. The experimental protocols were approved by the Seoul National University Institutional Animal Care and Use Committee (permission number SNU-170523-2), and all experiments were conducted according to the committee’s guidelines.

### AAV virus injection or drug treatment in the MASLD model

The HFD-, western diet-, and CDAA-high-fat/high-fat diet (HFD)-fed mouse models were described previously.^22,24,35^ To assess the function of *Wee1-AS*, an AAV encoding *Wee1-AS*, designated AAV-*Wee1-AS*, was constructed via the pAAV-MCS vector (VPK-410, Cell Biolabs, San Diego, CA, USA) with an AAV9 serotype, which includes the CMV promoter. For hepatocyte-specific expression, *Wee1-AS*, or GFP (as a control), was also constructed under a thyroxine-binding globulin (TBG) promoter and packaged with an AAV8 serotype, designated AAV-TBG-*Wee1-AS* or AAV-TBG-GFP. The AAV particles were assembled in HEK293T cells and purified via iodixanol gradient ultracentrifugation. The processes used to generate AAV-*Wee1-AS* and AAV-TBG-*Wee1-AS* were performed at the Virus Facility of the Korea Institute of Science and Technology (Seoul, South Korea) and Chonnam National University (Gwangju, South Korea), respectively. The mice were fed a LFD or HFD for 18 weeks. After 13 weeks of diet feeding, AAV-GFP or AAV-*Wee1-AS* (1 × 10^11^ genome copies/100 μl) was injected into the retro-orbital sinus. Although AAV9 is not hepatocyte specific, retro-orbital injection results in substantial accumulation in the liver.^36^ To investigate the effects of lncRNAs on hepatic fibrosis, seven-week-old mice were fed a CDAA-HFD (A06071306, Research Diets) or a control diet for seven weeks. After one week of diet feeding, AAV-TBG-GFP or AAV-TBG-*Wee1-AS* (8 × 10^11^ genome copies/100 μl) was injected into the retro-orbital sinus. After 13 weeks of diet feeding, adavosertib (HY-10993, MedChemExpress, Monmouth Junction, NJ, USA), which was suspended in 0.5% methyl cellulose, was administered daily at a dosage of 30 mg/kg/day by oral gavage for 5 weeks. All experiments were performed in a blinded and randomized fashion. At the end of feeding, liver tissues were excised, and small tissue blocks of the left lobe of the liver were fixed in 10% neutral buffered formalin or embedded in a formulation of glycols and resins.

### Analysis of free fatty acids in mouse serum

For the analysis of free fatty acids in mouse serum, 50 μL of mouse serum was mixed with 250 μL of methanol. The mixture was vortexed for 1 min, followed by shaking on the thermomixer at 4 °C for 1 h. Finally, 150 μL of extract was collected from the supernatant after centrifugation (4 °C, 20 min, 17,000 rcf), and a quality control (QC) sample was pooled from 15 μL of each extract. The QC sample was repeatedly injected and used for signal correction within the sequence.^37^ A general lipidomic analysis method was applied following the approach described by Cajka et al. (2016).^38^ A total of 5 μL of sample was injected into a 1290 UHPLC (Agilent) coupled with an Acquity UPLC CSH C18 column (100 × 2.1 mm, 1.7 μm) and a VanGuard precolumn (5 × 2.1 mm, 1.7 μm) (Waters, MA, USA). Data were acquired in scan mode via an Agilent Q-TOF 6530 mass spectrometer (MS). The acquired LC‒MS data were processed via MS-DIAL version 4.80 and statistically analyzed via MetaboAnalyst 6.0.^39,40^ Free fatty acid annotation was performed by comparing the m/z and retention times with those of fatty acid standards, as well as by referencing an in-house library. Statistical models were constructed via median normalization, log10 transformation, and Pareto scaling.

### Cell culture

Primary mouse hepatocytes were isolated from 8–10-week-old male C57BL/6 N mice as described previously.^41^ After perfusion, the cells were suspended in Dulbecco’s modified Eagle’s medium (DMEM) supplemented with 10% fetal bovine serum (FBS). Hepatic stellate cells (HSCs), liver sinusoidal endothelial cells (LSECs), and Kupffer cells were isolated from the nonparenchymal supernatant via a discontinuous Percoll gradient (52/50/30%; GE Healthcare, Waukesha, WI) and centrifuged at 2200 rpm. The top layer enriched with HSCs was collected. LSECs were isolated from the intermediate layer containing LSECs and Kupffer cells via magnetic-activated cell sorting with CD146 microbeads (#130-092-007; Miltenyi Biotech, Bergisch Gladbach, Germany) according to the manufacturer’s instructions. The remaining unbound fraction was collected as Kupffer cells. The purity of the isolated liver cell lines was validated by analyzing the expression of representative genes normalized to that of 18S rRNA (Supplementary Fig. 1). AML12 cells were obtained from the American Type Culture Collection and cultured in DMEM/F-12 medium supplemented with insulin–transferrin–selenium (Thermo Fisher Scientific, Waltham, MA, USA) and dexamethasone. Human primary hepatocytes (CHL-01, HLB, Sejong, South Korea) were cultured in DMEM/high-glucose medium supplemented with 10% FBS. Transient transfection of antisense oligonucleotides (ASOs) was performed via Lipofectamine 2000 (Invitrogen) according to the manufacturer’s protocol. Briefly, primary hepatocytes were seeded into culture plates. After 4 h, ASO-*Wee1-AS* (50 pmol) was transfected into the cells via Lipofectamine 2000 (Invitrogen). Following an additional 4 h of incubation, the medium was replaced with fresh medium containing 10% FBS, and the cells were incubated for 36 h. The ASO targeting *Wee1-AS*, ASO-*Wee1-AS*, was synthesized by Qiagen (Hilden, Germany) (Supplementary Table 4).

### scRNA sequencing, RNA sequencing, GEO database analysis

Mouse liver single-cell RNA sequencing (scRNA-seq) data were obtained from the NCBI Gene Expression Omnibus (GEO) under accession GSE156057, with a focus on the 24-week group with matched Cd45+ and Cd45- populations under LFD- and WD-fed conditions.^42^ Raw reads were retrieved via the SRA Toolkit v2.11.3 and processed via Cell Ranger v8.0.1 to generate a cell-count matrix via GENCODE release M23 (GRCm38.p6 genome assembly) as the reference annotation. *Wee1-AS* transcript information was incorporated into the reference for quantification. Low-quality cells were excluded from the cell-count matrix by applying quality control filters.^43^ Downstream analysis was performed via Seurat v5.0.3, with batch effects corrected via the FindIntegrationAnchors function.^44^ Dimensionality reduction was conducted via principal component analysis (PCA) and uniform manifold approximation and projection (UMAP) on variable genes. Cell types were identified on the basis of Liver Cell Atlas (livercellatlas.org) annotations.^42^

For global RNA sequencing, a cDNA library was independently prepared with poly-A-containing mRNA molecules with reverse transcriptase, DNA Polymerase I, RNase H, and dUTP. After the products were purified and enriched via PCR to create the final cDNA library, the final cDNA libraries were quantified and qualified. Indexed libraries were then submitted to Illumina NovaSeq (Illumina, Inc., San Diego, CA, USA), and paired-end (2 × 100 bp) sequencing was performed by Macrogen Inc. (Seoul, South Korea).

Human liver bulk RNA-seq datasets from NCBI GEO were analyzed: GSE126848 and GSE162694.^45,46^ Transcript-level quantification was performed on adaptor-trimmed FASTQ reads via Salmon v1.10.1 in quasimapping mode, with a custom reference index built from GENCODE release 44 (GRCh38.p14) and the LNC106435.1 transcript. Transcript abundance was normalized to transcripts per million (TPM) and aggregated via the tximport R package v1.32.0. To mitigate batch effects across samples, we used reciprocal principal component analysis-based integration. In brief, 1000 variable features were selected per sample via the default parameters of the *SelectIntegrationFeatures* function. Integration anchors were then computed via the *FindIntegrationAnchors* function with the top 20 principal components and k.anchor = 5. Both the Cd45+ and Cd45- cell populations under LFD conditions were used as reference samples for alignment. The datasets were then aligned via *IntegrateData*, and dimensionality reduction was performed on the integrated data via principal component analysis and uniform manifold approximation and projection.

### RACE and fluorescence in situ hybridization

The 5′ and 3′ RACE-ready cDNAs for *Wee1-AS* were amplified via a GeneRacer^TM^ Kit (L1502-01, Invitrogen). RACE and nested RACE PCRs were performed with Biotechnology i-StarTaq^TM^ DNA Polymerase (iNtRON Biotechnology, Seongnam, South Korea). The resulting RACE PCR products were subsequently cloned via the TOPO TA cloning kit (Invitrogen) and sequenced. For RACE of *LNC106435.1*, the SMARTer RACE 5′/3′ Kit (Takara Bio, Otsu, Japan) was used. RACE and nested RACE PCRs were performed with SeqAmp DNA Polymerase (638504, Takara Bio).

For FISH, frozen liver sections were fixed and dehydrated by ethanol according to the manufacturer’s protocol for the RNAscope^TM^ Multiplex Fluorescent Reagent Kit v2 (ACD Bio, Newark, CA, USA). After peroxidation, the sections were treated with protease IV for 30 min, after which the *Wee1-AS* probe or control probe was hybridized with the sections. After sequential amplification of fluorescence, the sections were mounted and examined via a confocal microscope. The specificity of the FISH results was validated by the disappearance of the FISH signal after *Wee1-AS* was knocked down in hepatocytes. In addition, positive control (*Polr2a*) and negative control (*dapB*) data are shown (Supplementary Fig. 10).

### Subcellular fractionation

For subcellular fractionation, primary hepatocytes were suspended in buffer containing 0.15% NP-40, 10 mM Tris-HCl (pH 7.0), and 150 mM NaCl. After adding 25% sucrose slowly, the mixture was centrifuged, and the supernatant was saved as the cytoplasmic fraction. The precipitated nuclear pellet was washed and resuspended in 50% glycerol buffer. After the nuclei were lysed in buffer containing 1% NP-40 and 1 M urea and centrifuged, the supernatant was collected as the nucleoplasm fraction. The remaining chromatin pellet was washed with cold PBS and saved as the chromatin fraction. Easy blue (iNtRON Biotechnology, Seongnam, South Korea) was added to each fraction to extract RNA.

Mitochondria were isolated from primary hepatocytes via a mitochondria isolation kit (89874; Thermo Scientific, Waltham, MA, USA). A proteinase K protection assay was performed to examine the submitochondrial distribution of proteins. Isolated mitochondria were suspended in TD buffer (10 mM HEPES, pH 8.0; 250 mM sucrose; 0.5 mM EGTA) and incubated with proteinase K at RT. The digestion was terminated by the addition of PMSF, and the remaining proteins were analyzed by SDS‒PAGE/western blotting.

### Quantitative real-time PCR (qRT‒PCR) and RNA pull-down assay

The mRNA expression of genes was determined by qRT‒PCR via an ABI StepOnePlus^TM^ Real-time PCR system (Applied Biosystems, Foster City, CA, USA) with specific primers (Supplementary Table 2). Relative mRNA expression was calculated relative to that of the controls via the 2^–ΔΔCT^ method.^41^ For the RNA pull-down assay, *Wee1-AS* and its antisense strand were synthesized in vitro via the MEGAscript T7/SP6 Transcription Kit (Invitrogen) and biotinylated with a Biotin RNA Labeling mix (Roche, Basel, Switzerland). After purification and proper folding, 15 pmol of folded RNA was added to the precleared liver tissue lysates and pulled down with streptavidin-conjugated agarose beads. The resulting pulled-down proteins were subjected to gel electrophoresis and visualized through silver staining. Differentially expressed protein bands were cut and analyzed via mass spectrometry.

### Western blotting, immunohistochemistry, immunofluorescence, and chromatin immunoprecipitation (ChIP)‒qPCR

Western blotting was performed as previously described using specific antibodies.^47^ The intensity of the protein bands was quantified via ImageJ software. For immunohistochemistry, 10 μm sections of paraffin-embedded tissue were stained with specific antibodies. Stained tissues were examined via an automated multimodal tissue analysis system. For immunofluorescence, primary hepatocytes were fixed with ice-cold methanol and stained with specific antibodies as previously described.^35^ The stained samples were then examined via a confocal microscope. The ChIP‒qPCR assay was conducted as described previously with specific antibodies.^48^ The immunoprecipitated genome region was amplified via the SYBR Green Master mix (Applied Biosystems, Foster City, CA, USA) with specific primers (Supplementary Table 2). The data were normalized to the input and analyzed relative to the nonspecific IgG control.

### Assessment of mitochondrial function, the oxygen consumption rate, and lipid accumulation

For SDH staining, sections of frozen liver tissues or primary hepatocytes were incubated in 50 mM sodium phosphate-buffered solution (pH 7.6) containing 50 mM sodium succinate and 0.6 mM nitrotetrazolium blue chloride at 37 °C. After 30 min, the samples were washed and examined via an automated multimodal tissue analysis system (Vectra 3, PerkinElmer, Waltham, MA, USA). To determine the mitochondrial mass, primary hepatocytes were stained with a MitoTracker Green FM probe (Invitrogen). To assess mitochondrial function, hepatocytes were stained with MitoTracker Red CMXRos (Invitrogen), or the cells were stained with tetramethylrhodamine methyl and ethyl esters (TMRM) to assess the mitochondrial membrane potential. Mitochondrial superoxide production was evaluated via the use of MitoSOX dye (Invitrogen). The stained samples were examined via a confocal microscope. Electron microscopy was performed as previously described.^48^ Briefly, whole liver samples were fixed with osmium tetroxide, followed by en bloc staining with 0.5% uranyl acetate. The samples were then dehydrated using ethanol, embedded in Spurr’s resin, and incubated at 70 °C for polymerization of the resin. Ultrathin sections were cut via an ultramicrotome and examined via transmission electron microscopy (JEOL, Tokyo, Japan).

The oxygen consumption rate (OCR) was measured by using an XF96e Extracellular Flux analyzer (Agilent Technologies). Mouse primary hepatocytes (5 × 10^3^ cells per well) were directly plated in poly-D-lysine-coated XF96 cell culture plates, and ASO-*Wee1-AS* was transfected after 4 h. The next day, palmitic acid (10 μM) and adavosertib were added for 18 h and 24 h, respectively. Two days after seeding, the growth medium in the wells was replaced with XF running medium (XF DMEM supplemented with 4.5 g/L glucose, 1 mM sodium pyruvate, and 4 mM L-glutamine), and the plate was transferred to a 37 °C CO_2_-free incubator for 1 h. For the OCR measurement, carbonyl cyanide-4-(trifluoromethoxy)phenylhydrazone (FCCP, an inducer of maximal respiration; 0.5 μM) and rotenone/antimycin A (0.5 μM) were added, and 3 baseline measurements were taken prior to the addition of any compound, and 3 response measurements were taken after the addition of each compound.

To determine lipid accumulation, primary hepatocytes were fixed with 4% formaldehyde solution and stained with 5 μM BODIPY (D3922; Thermo Fisher Scientific) at room temperature for 30 min. Stained hepatocytes were examined via a confocal microscope.

### Human MASLD liver tissue analysis

Liver tissue samples from normal, metabolic dysfunction-associated steatotic liver (MASL), and MASH patients were obtained from the Seoul Metropolitan Government Boramae Medical Center (Seoul, Korea). Details of the patient cohorts are described in Supplementary Table 2. The samples were sectioned, and immunohistochemistry was performed.

### Statistical analysis

The number of samples is indicated in the figure legends, and three or four independent biological experimental replicates were conducted. All analyses were performed with GraphPad Prism software (GraphPad Software, San Diego, CA, USA). Statistical analyses between two groups were conducted via the nonparametric Mann–Whitney *U* test (two-tailed). When the experiments included more than two groups, differences between groups were analyzed via one-way ANOVA. Two-way ANOVA was used to compare the means of two independent variables or factors from two or more populations. The data are presented as the means ± SDs. Statistical significance was set at *P* < 0.05.

## Supplementary information

Supplementary_Materials

Uncropped western blots

## Data Availability

The raw RNA sequencing data used in the present study have been submitted to the Gene Expression Omnibus (GEO) and can be found under the accession number GSE289618 (https://www.ncbi.nlm.nih.gov/geo/query/acc.cgi?acc=GSE289618).
