# Supplementary material for: LncRNA Wee1-AS coordinates oxidative fatty acid metabolism through the activation of mitochondrial CDK1/CYCLIN B1
Source: Signal Transduct Target Ther. 2026 Jan 10;11:13. doi: 10.1038/s41392-025-02558-4 (PMC12790571; doi:10.1038/s41392-025-02558-4)
Supplement: Supplementary file 1 — Supplementary_Materials [file 41392_2025_2558_MOESM1_ESM.docx]

Supplementary Materials for

LncRNA *Wee1-AS* coordinates oxidative fatty acid metabolism through the activation of mitochondrial CDK1/CYCLIN B1

Hyeon-Ji Kim^#^, Cheolhee Jeong^#^, Sang-Heon Lee, Seungchan An, Gyu Hwan Hyun, Ga Young Lim, Ju-Yeon Kim, Junhyeong Lee, Min-Jung Park, Sung Won Kwon, Won Kim, Minsoo Noh, Yong-Hyun Han, and Mi-Ock Lee

Correspondence to: molee@snu.ac.kr

**This PDF file includes:**

Materials and Methods

Figures. S1 to S10

Tables S1 to S4

**Other Supplementary Materials for this manuscript include the following:**

Uncropped western blots

Materials and Methods

Measurement of half-life of *Wee1-AS* in primary mouse hepatocytes

To evaluate the stability of *Wee1-AS*, primary mouse hepatocytes were treated with actinomycin D (1 µg/mL; A9415, Sigma-Aldrich) to inhibit *de novo* transcription. Cells were harvested at the indicated time points (0, 1, 2, 4, 8, and 18 h) after actinomycin D treatment. The relative abundance of *Wee1-AS* transcripts at each time point was quantified by qRT–PCR and was expressed as a percentage of the value at 0 h. The decay curve was fitted using a one-phase exponential decay model in GraphPad Prism to determine the half-life of the transcript.

Knockdown of Wee1 in the mouse MASLD model

To assess the function of Wee1, a liver-specific AAV vector expressing short hairpin RNA (shRNA) targeting *Wee1* was generated. Two different target sequences were incorporated into a single pAAV-dual miR30-shRNA construct under the control of the hepatocyte-specific thyroxine-binding globulin (TBG). A scramble control containing two different non-targeting sequences was constructed using the same vector (Supplementary Table 4). Both constructs were designed to co-express EGFP independently and were packaged into AAV8 serotype capsids, yielding AAV-TBG-shWee1 or AAV-TBG-shScramble, respectively. The AAV particles were assembled in HEK293T and purified using iodixanol gradient ultracentrifugation. Seven-week-old mice were injected *via* the retro-orbital sinus with either AAV-TBG-shScramble or AAV-TBG-shWee1 (8 x 10^11^ genome copies/100 μl). After two weeks of virus injection, mice were fed with CDAA-HFD (A06071306, Research Diets) or control diet for four weeks. All experiments were performed in a blinded and randomized fashion. The experimental protocols were approved by the Seoul National University Institutional Animal Care and Use Committee (permission number SNU-220211-1 and SNU-250527-2) and all experiments were conducted according to the committee’s guidelines.

Figure. S1.


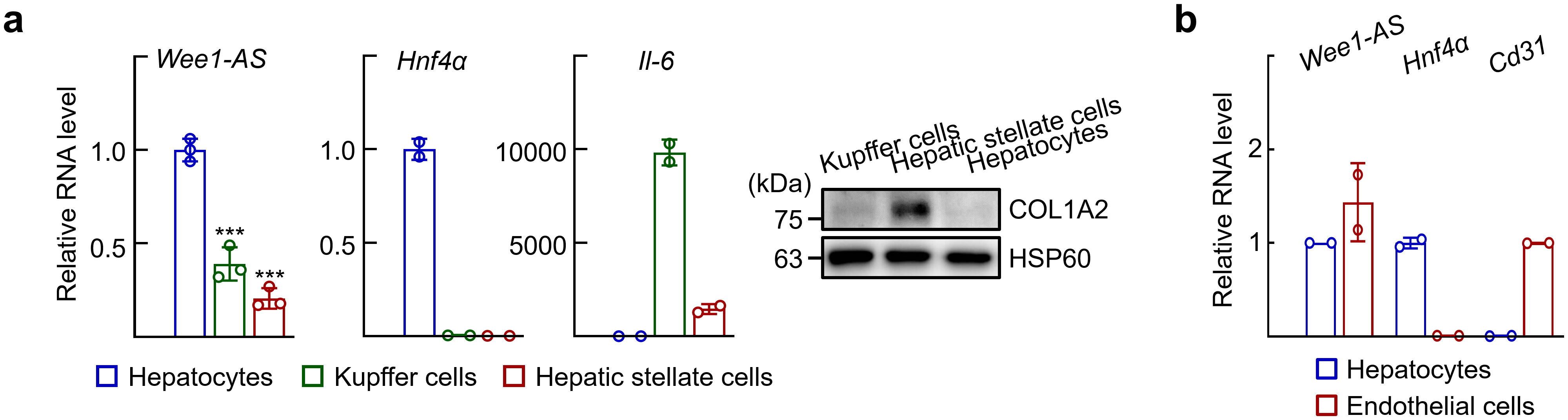


**Supplementary Fig. 1: *Wee1-AS* expression in hepatocytes, Kupffer cells, hepatic stellate cells, and liver sinusoidal endothelial cells.**

**a,** The relative RNA levels of *Wee1-AS* and representative markers of hepatocytes and Kupffer cells were measured across three distinct cell populations in liver. The expression of COL1A2 protein (hepatic stellate cell marker) was analyzed via western blotting across three distinct cell populations in liver. The values are represented the means ± SDs (n=3). The data were analyzed via one-way ANOVA. ^***^*P* < 0.001 *vs* Hepatocytes.

**b,** The relative RNA levels of *Wee1-AS*, *Hnf4a* (hepatocyte marker), and *Cd31* (endothelial cell marker) were measured via qRT-PCR in hepatocytes and liver sinusoidal endothelial cells.

Figure. S2.


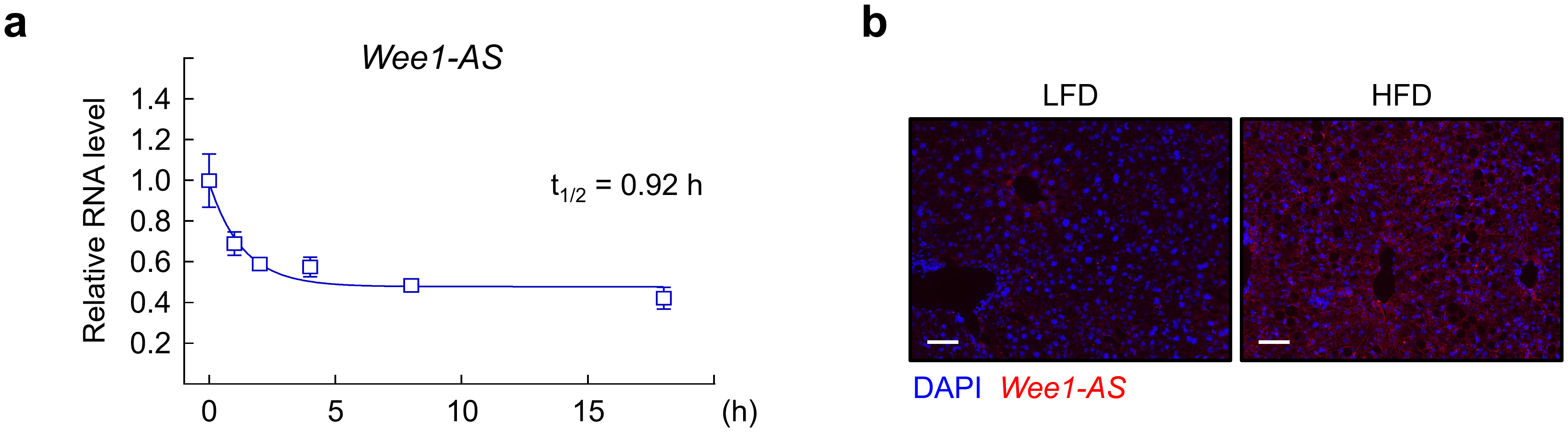


**Supplementary Fig. 2: Half -life of *Wee1-AS* in primary mouse hepatocytes and FISH signals of *Wee1-AS* in the liver tissues of LFD or HFD-fed mice.**

**a,** Primary mouse hepatocytes were treated with actinomycin D (1 µg/mL) to block *de novo* transcription and *Wee1-AS* levels were measured by qRT–PCR at the indicated times (0, 1, 2, 4, 8, 18 h). The decay curve was fitted to a one-phase exponential model in GraphPad Prism, yielding a half-life of approximately 0.92 h. The values are represented the means ± SDs (n=4).

**b,** Fluorescence in situ hybridization (FISH) analysis was performed in liver tissues from mice fed a low-fat diet (LFD) or high-fat diet (HFD). Scale bar, 50 μm.

Figure. S3.


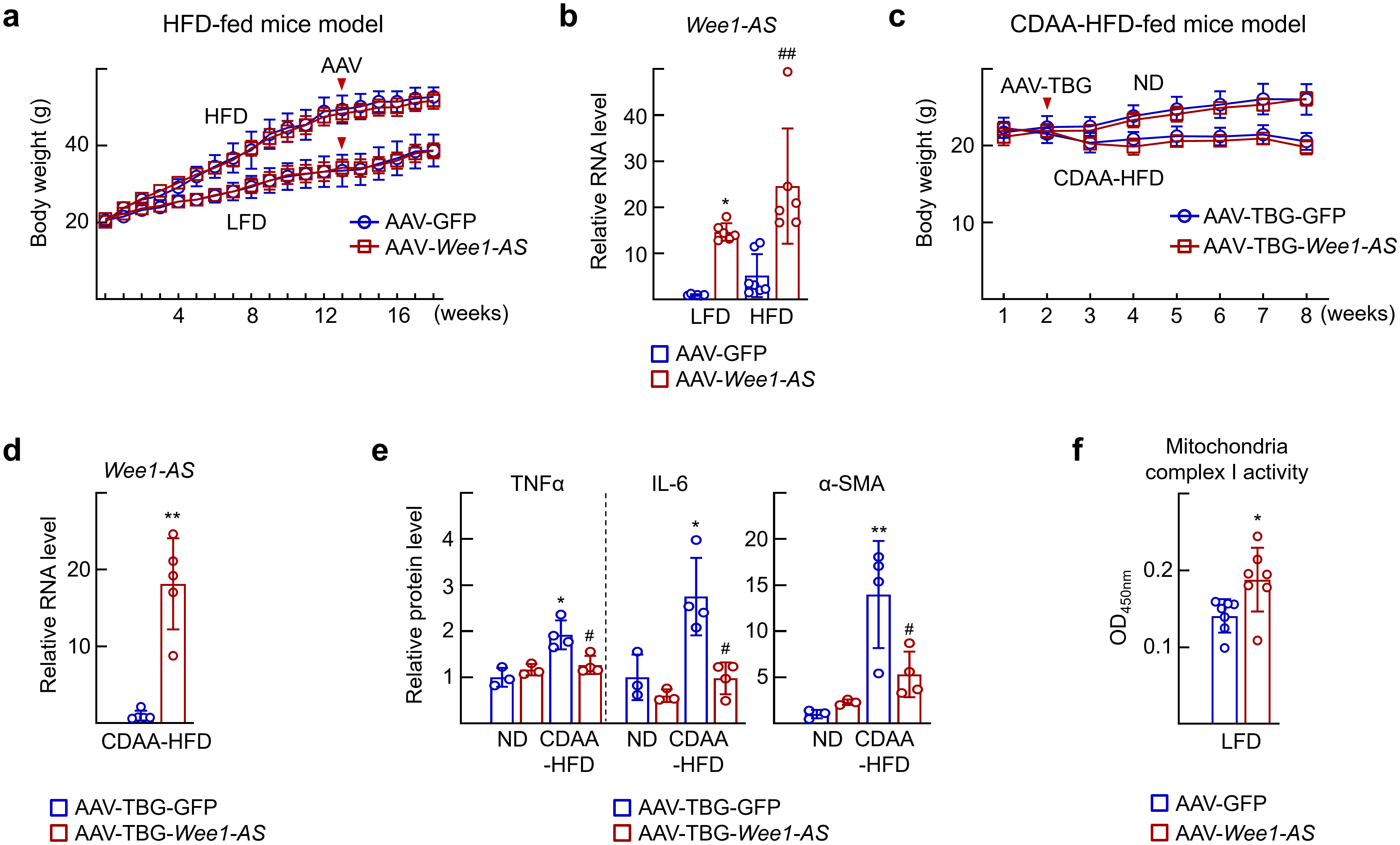


**Supplementary Fig. 3: AAV-*Wee1-AS* and AAV-TBG-*Wee1-AS* transduction in HFD- and CDAA-HFD-fed mice models.**

**a,** Body weight change curve of seven-week-old C57BL/6 N mice fed with either low-fat diet (LFD) or high-fat diet (HFD) for 18 weeks. At 13 weeks, AAV-*Wee1-AS* was transfused retro-orbitally.

**b,** Relative RNA levels of *Wee1-AS* in LFD- and HFD-fed mice following AAV-GFP or AAV-*Wee1-AS* infusion, measured via qPCR. The values are represented the means ± SDs (n=6-7). The data were analyzed via two-way ANOVA. ^*^*P* < 0.05 *vs* LFD with AAV-GFP; ^##^*P* < 0.01 *vs* HFD with AAV-GFP.

**c,** Body weight change curve of seven-week-old C57BL/6 N mice fed with either normal diet (ND) or choline-deficient, L-amino acid-defined high-fat diet (CDAA-HFD) for 7 weeks. At 1 week, AAV-TBG-*Wee1-AS* was transfused retro-orbitally.

**d,** Relative RNA levels of *Wee1-AS* in CDAA-HFD-fed mice following AAV-TBG-GFP or AAV-TBG-*Wee1-AS* infusion, measured via qPCR. The values are represented the means ± SDs (n=5). ^**^*P* < 0.05 *vs* CDAA-HFD with AAV-TBG-GFP.

**e,** Expression of TNFα, IL-6, and α-SMA in liver tissues was analyzed via western blotting. The values are represented the means ± SDs (n=3-4). ^*^*P* < 0.05 and ^**^*P* < 0.01 *vs* ND with AAV-TBG-GFP; ^#^*P* < 0.05 *vs* CDAA-HFD with AAV-TBG-GFP.

**f,** Activities of mitochondrial complex I in liver tissues from LFD-fed mice shown in Fig. 2a were measured via spectrophotometry based on the rates of NADH oxidation. The values are represented the means ± SDs (n=7). The data were analyzed via two-way ANOVA. ^*^*P* < 0.05 *vs* LFD with AAV-GFP.

Figure. S4.


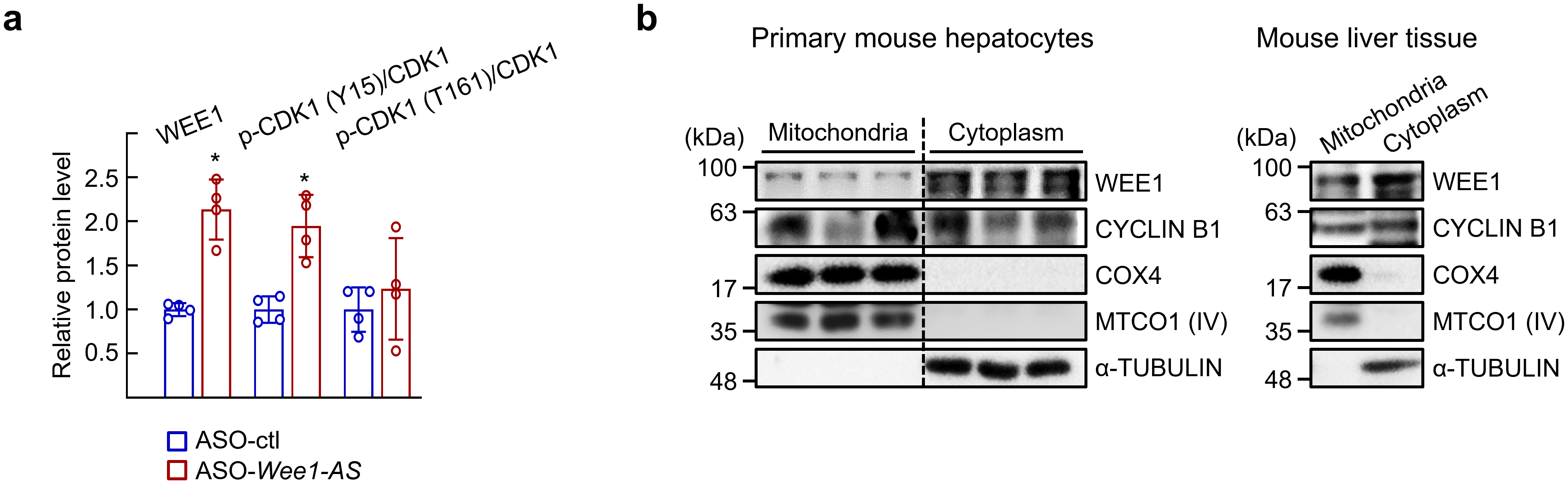


**Supplementary Fig. 4: Analysis of WEE1/p-CDK1 expression and validation of subcellular fractionation.**

**a,** Expression of the indicated proteins was analyzed via western blotting. Intensity of each protein band was quantified via ImageJ and normalized to that of β-ACTIN or CDK1. ^*^*P* < 0.05 *vs* ASO-control (ctl).

**b,** Subcellular fractionation of primary mouse hepatocytes (left) and liver tissues (right) was performed and was validated via western blot analysis, showing efficient separation of mitochondrial (COX4 and MTCO1) and cytoplasmic (α-TUBULIN) markers.

Figure. S5.


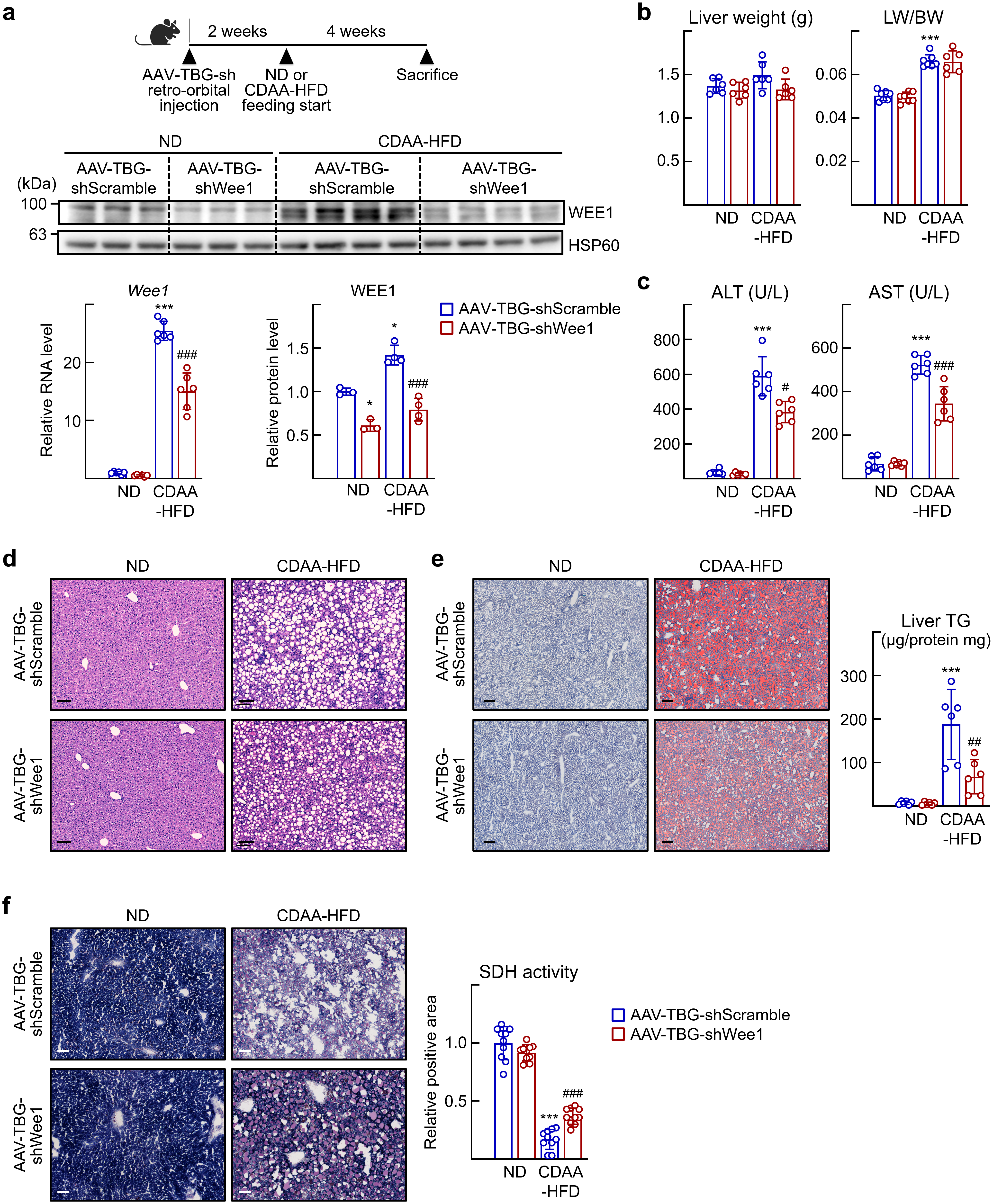


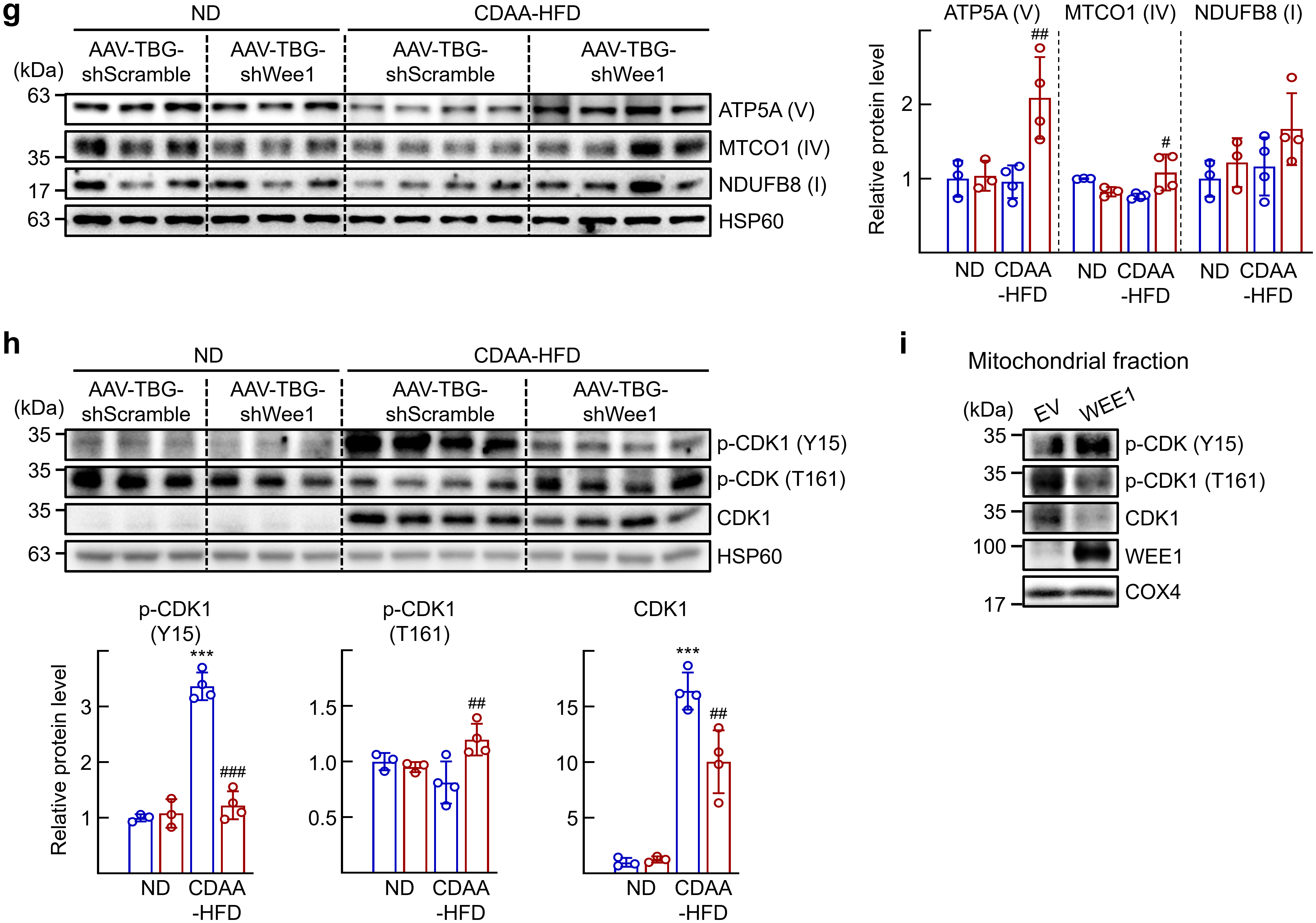


**Supplementary Fig. 5: Knockdown of *Wee1* improves symptoms of MASLD in the CDAA-HFD-fed mice.**

**a,** Seven-week-old mice were injected via the retro-orbital sinus with either AAV-TBG-sh-Scramble or AAV-TBG-shWee1. After two weeks of virus injection, mice were fed with choline-deficient, L-amino acid-defined high-fat diet (CDAA-HFD) or normal diet (ND) for 4 weeks. The RNA and protein levels of Wee1 were analyzed via qRT–PCR or western blotting. ^*^*P* < 0.05, ^**^*P* < 0.01, and ^***^*P* < 0.001 *vs* ND with AAV-TBG-shScramble; ^###^*P* < 0.001 *vs* CDAA-HFD with AAV-TBG-shScramble.

**b,** Liver weights and liver weight/body weight (LW/BW) ratios at the end of the experiments are shown. The values are represented the means ± SDs (n=6). The data were analyzed via two-way ANOVA. ^***^*P* < 0.001 *vs* ND with AAV-TBG-shScramble.

**c,** Serum ALT and AST activities were measured via standard clinical chemistry assays. The values are presented the means ± SDs (n=6). The data were analyzed by two-way ANOVA. ^***^*P* < 0.001 *vs* ND with AAV-TBG-shScramble; ^#^*P* < 0.05 and ^###^*P* < 0.001 *vs* CDAA-HFD with AAV-TBG-shScramble.

**d,** H&E staining of liver sections. Scale bar: 100 μm.

**e,** Oil red O staining of liver sections and hepatic TG levels. Scale bar, 200 μm. The values are represented the means ± SDs (n=6). The data were analyzed by two-way ANOVA. ^***^*P* < 0.001 *vs* ND with AAV-TBG-shScramble; ^##^*P* < 0.01 *vs* CDAA-HFD with AAV-TBG-shScramble.

**f,** Representative images of SDH activity staining in the liver sections. Scale bar, 100 μm. The intensity was quantified in 2 images for a liver tissue using ImageJ. The data were analyzed by two-way ANOVA. ^***^*P* < 0.001 *vs* ND with AAV-TBG-shScramble. ^###^*P* < 0.001 *vs* CDAA-HFD with AAV-TBG-shScramble.

**g,** Hepatic levels of proteins in electron transport chain (ETC) complexes were analyzed via western blotting using a commercially available anti-total OXPHOS primary antibody cocktail. ATP5A, ATP synthase, H+ transporting, mitochondrial F1 complex, alpha 1; MTCO1, mitochondrially encoded cytochrome c oxidase I; NDUFB8, NADH dehydrogenase [ubiquinone] 1 beta subcomplex subunit 8. Roman numbers represent the corresponding ETC complex. Band intensities of each protein were quantified via ImageJ. The values are presented as the means ± SDs (n=3-4). ^#^*P* < 0.05 and ^##^*P* < 0.01 *vs* CDAA-HFD with AAV-TBG-shScramble.

**h,** The expression of CDK1 proteins of liver tissues was analyzed via western blotting. The values are represented the means ± SDs (n=3-4). ^***^*P* < 0.001 *vs* ND with AAV-TBG-shScramble; ^##^*P* < 0.01 and ^###^*P* < 0.001 *vs* CDAA-HFD with AAV-TBG-shScramble.

**i,** Primary hepatocytes were transfected with Flag-empty vector (EV) or Flag-WEE1 (WEE1) and were treated with 25 μM palmitic acid-conjugated with 0.05% bovine serum albumin. The mitochondrial fraction was prepared and analyzed via western blotting.

Figure. S6.


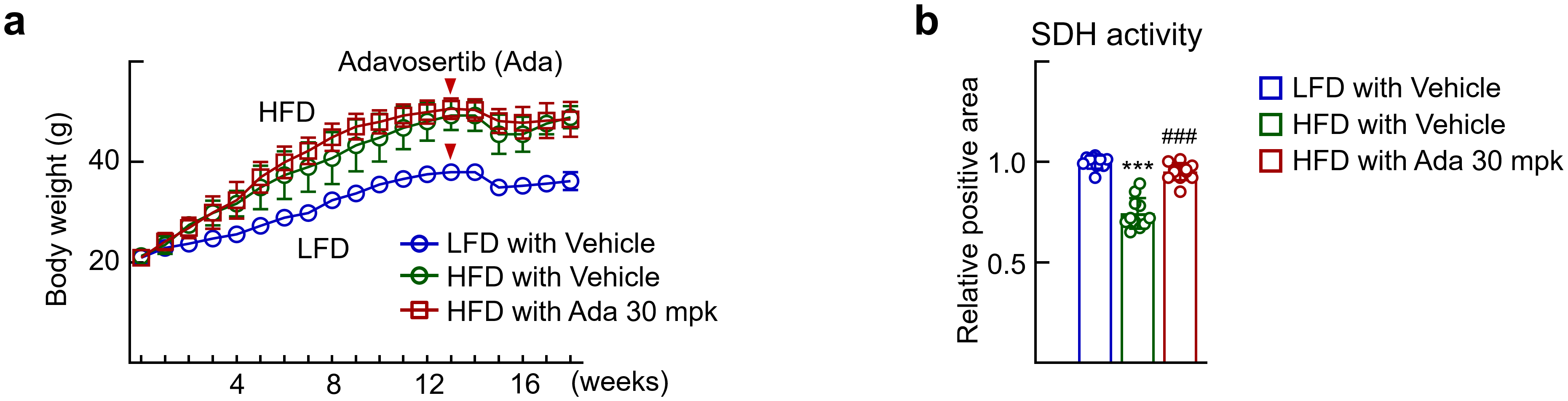


**Supplementary Fig. 6: HFD-fed and adavosertib-treated mouse model.**

**a,** Body weight change curve of seven-week-old C57BL/6 N mice fed with either low-fat diet (LFD) or high-fat diet (HFD) for 18 weeks. At 13 weeks, adavosertib (30 mg/kg body weight) or vehicle (0.5% methylcellulose) was orally administered daily for 5 weeks.

**b,** The SDH activity was quantified in 2 images for a liver tissue using ImageJ. The data were analyzed by one-way ANOVA. ^***^*P* < 0.001 *vs* LFD with vehicle; ^###^*P* < 0.001 *vs* HFD with vehicle.

Figure. S7.


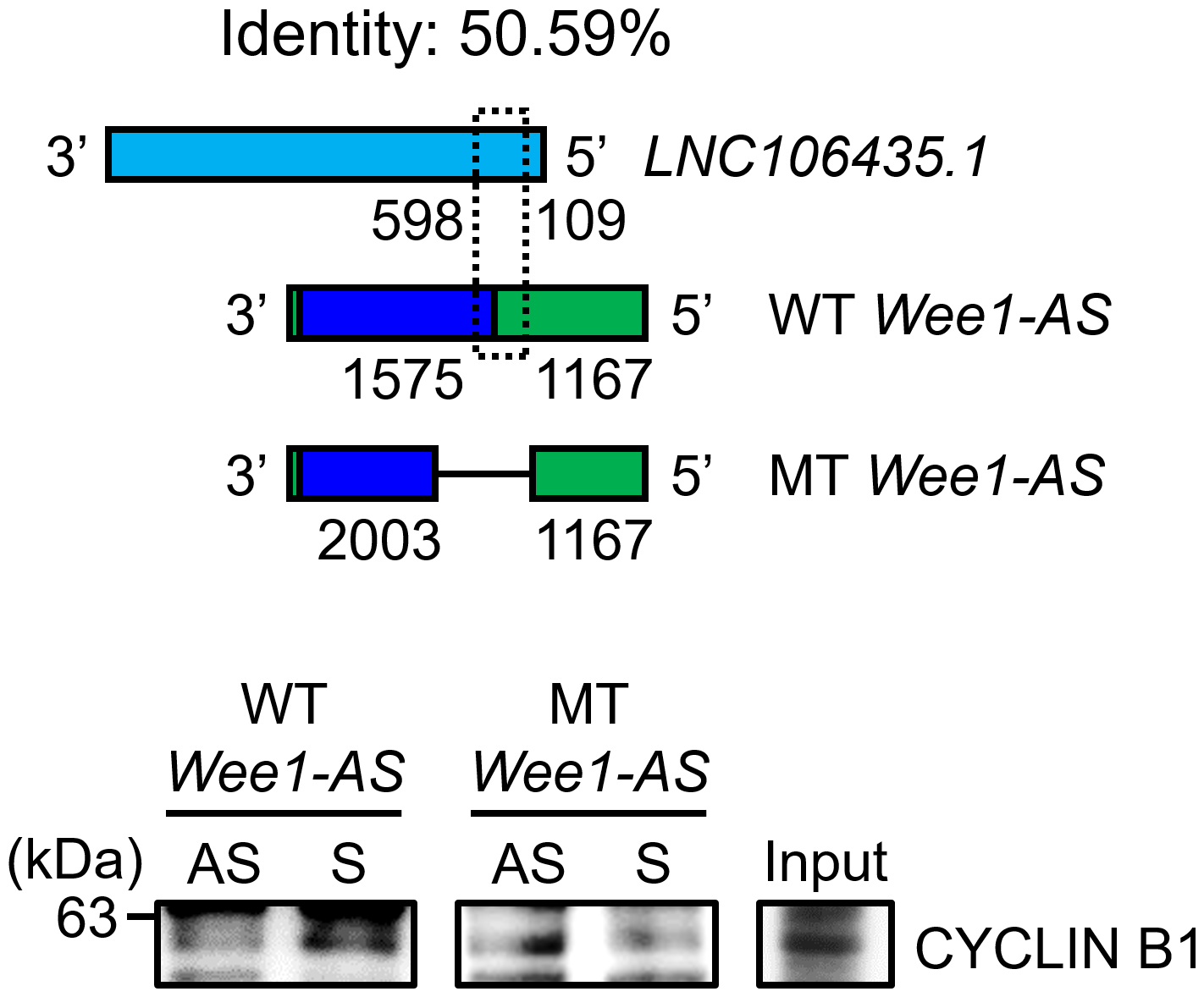


**Supplementary Fig. 7: CYCLIN B1 bound to WT *Wee1-AS*, but not to MT *Wee1-AS*.**

RNA pull-down assays were performed using biotinylated wild-type (WT) *Wee1-AS* (sense, S) or its antisense (AS), and mutant (MT) *Wee1-AS* lacking the conserved sequence (1167–2003 nt) (S) or its antisense (AS), with protein lysates prepared from mouse liver tissues. Pull-downed proteins were analyzed by Western blotting.

Figure. S8.


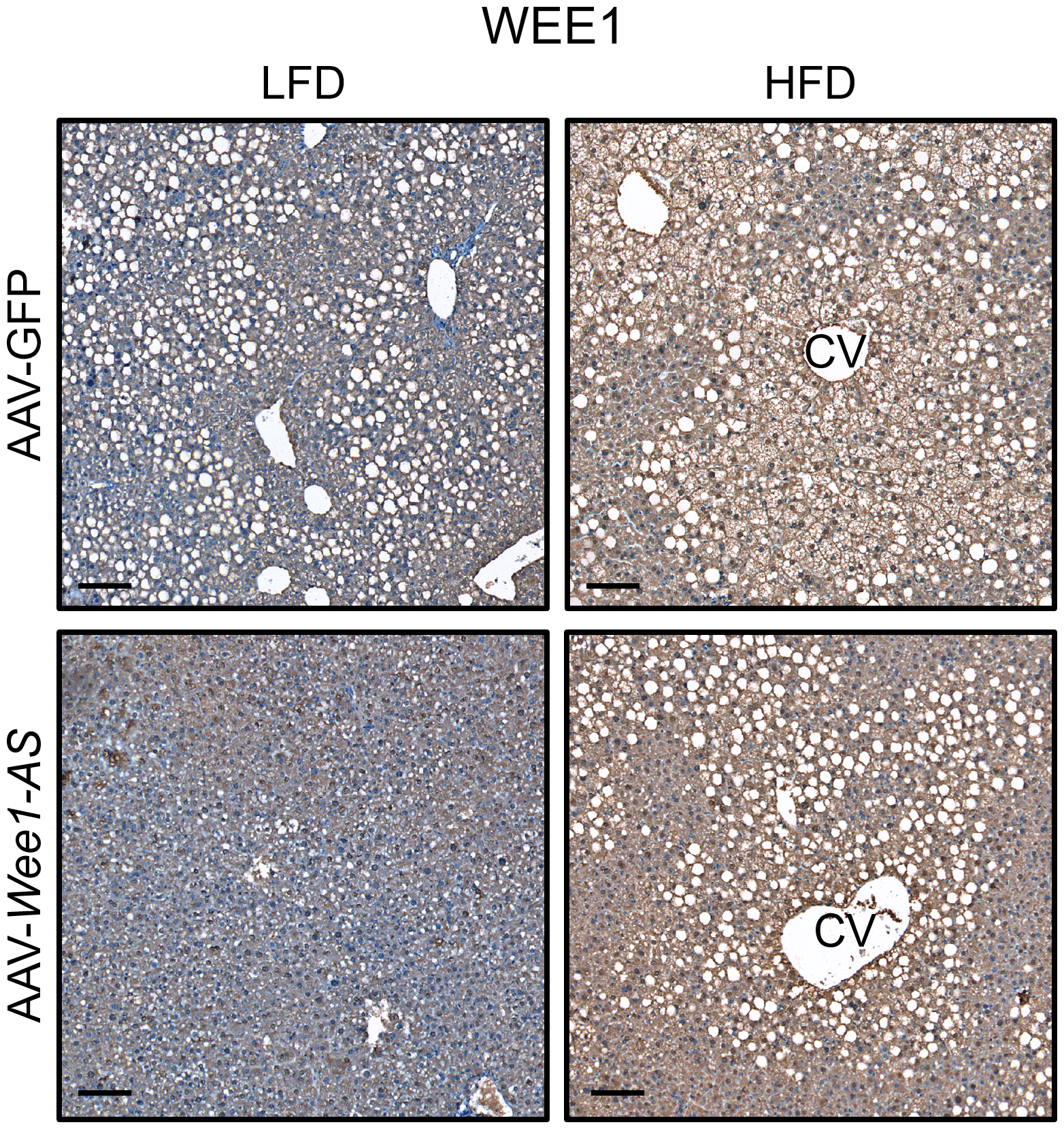


**Supplementary Fig. 8: Immunohistochemistry of WEE1 in the liver tissues.**

Representative images of immunohistochemical staining of WEE1 in the liver sections of mice shown in Fig. 2a. Scale bar, 100 μm.

Figure. S9.


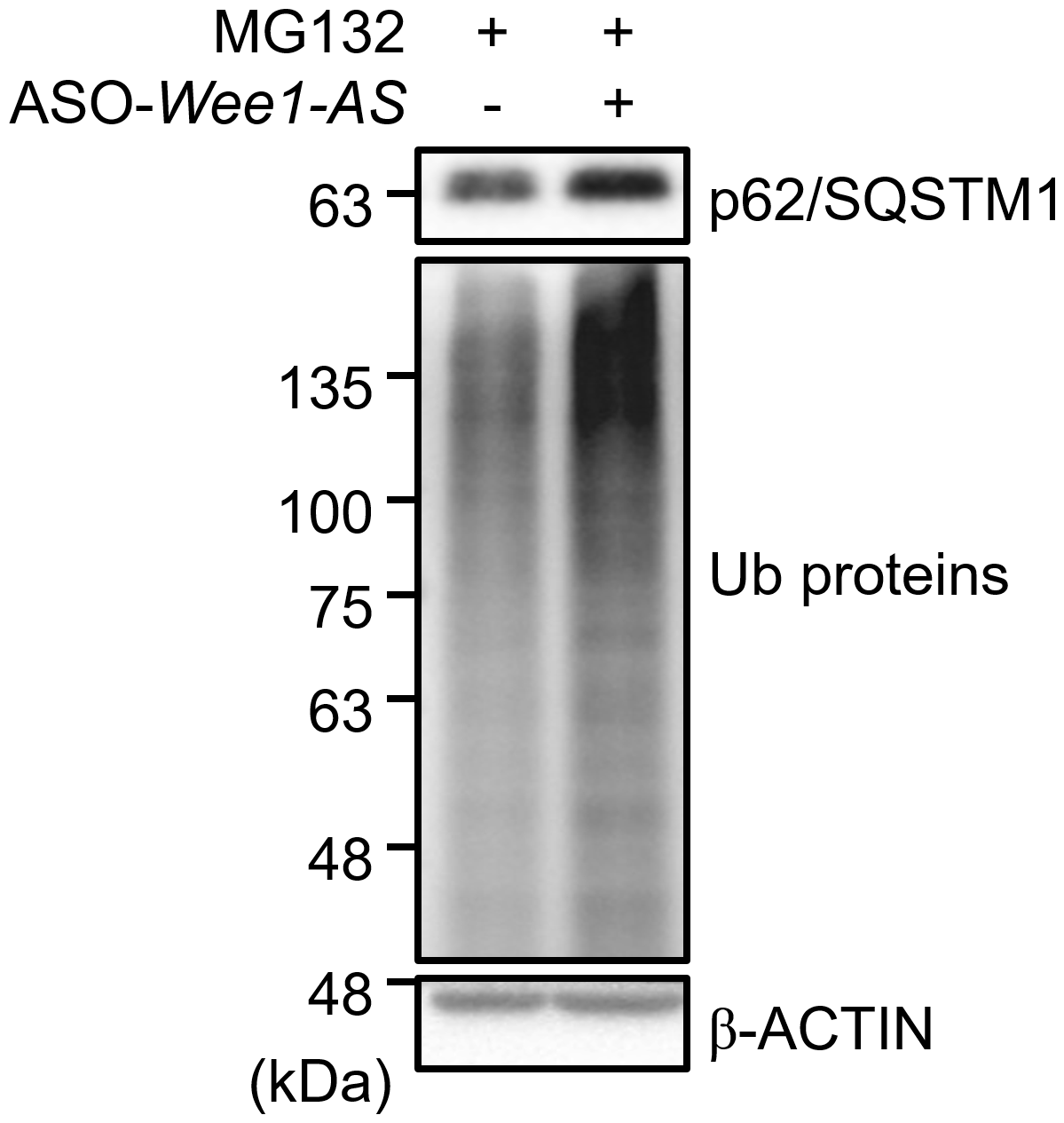


**Supplementary Fig. 9: The expression of p62/SQSTM1 and ubiquitinated proteins in *Wee1-AS* knockdown**

Primary hepatocytes were transfected by ASO-control or ASO-*Wee1-AS* and treated with 20 μM MG132 for 3 h. Then, the expression of indicated proteins was analyzed via western blotting.

Figure. S10.


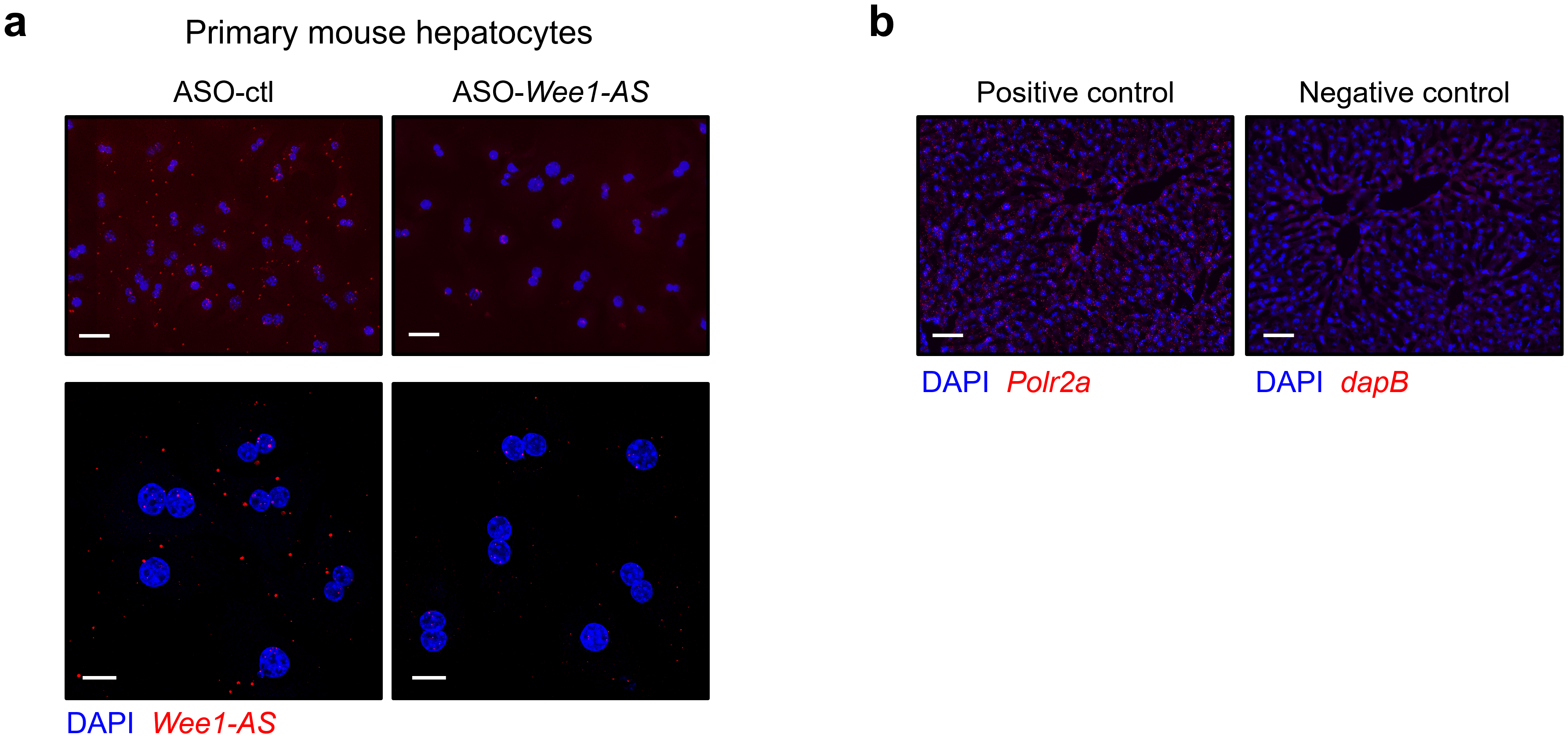


**Supplementary Fig. 10: Validation of *Wee1-AS* FISH signals in primary hepatocytes and liver tissues.**

**a,** Fluorescence in situ hybridization (FISH) analysis was performed in primary hepatocytes following transfection of ASO-control (ctl) or ASO-*Wee1-AS*. Scale bar, 50 μm (top); 20 μm (bottom).

**b,** *Polr2a* and *Dapb* were used as positive and negative controls, respectively in normal liver tissues. Scale bar, 50 μm.

Table S1.

| **Transcript ID** | **Gene symbol** | **Location** | **Length** | **Fold Change** | | |
| --- | --- | --- | --- | --- | --- | --- |
|  |  |  |  | **HM/LM** | **HF/LF** | **HO/LO** |
| ENSMUST00000159927 | Themis | chr10 | 4827 | 83.65 | 1.66 | 27.04 |
| AK050408 | AK050408 | chr5 | 1845 | 10.56 | 1.09 | 2.62 |
| uc009ilc.1 | AK031221 | chr7 | 2760 | 9.54 | 1.47 | 2.41 |
| uc009bxa.1 | Osbpl3 | chr6 | 3218 | 9.00 | 1.07 | 7.33 |
| uc029tiw.1 | Plin4 | chr17 | 4562 | 8.00 | 1.30 | 9.35 |
| AK039872 | AK039872 | chr2 | 2543 | 6.24 | 1.86 | 3.37 |
| NR_045778 | C730002L08Rik | chr19 | 2917 | 6.23 | 1.77 | 2.78 |
| ENSMUST00000124848 | Gm13074 | chr4 | 2735 | 6.16 | 1.32 | 3.43 |
| ENSMUST00000125577 | Gm11724 | chr11 | 472 | 5.60 | 1.33 | 2.16 |
| AK081893 | AK081893 | chr7 | 1754 | 5.54 | 1.24 | 2.13 |
| ENSMUST00000140421 | Gm15627 | chr5 | 437 | 5.20 | 1.30 | 2.24 |
| ENSMUST00000143291 | Gm11752 | chr11 | 510 | 5.10 | 1.67 | 2.02 |
| NR_077233 | C230037L18Rik | chr15 | 872 | 4.78 | 1.04 | 2.11 |
| TCONS_00010915 | XLOC_008385 | chr15 | 1700 | 4.74 | 1.92 | 2.46 |
| NR_040428 | Gm19619 | chr5 | 2856 | 4.72 | 1.20 | 2.06 |
| TCONS_00010916 | XLOC_008385 | chr15 | 1515 | 4.61 | 1.76 | 2.88 |
| NR_015502 | E030003E18Rik | chr19 | 1085 | 4.59 | 1.43 | 2.52 |
| TCONS_00036025 | XLOC_026627 | chrX | 2219 | 4.20 | 1.51 | 3.05 |
| ENSMUST00000117514 | Gm7856 | chr16 | 1493 | 3.81 | 1.12 | 2.07 |
| TCONS_00010929 | XLOC_008416 | chr15 | 1637 | 3.52 | 0.97 | 2.96 |
| NR_045698 | E330011O21Rik | chr16 | 950 | 3.39 | 1.26 | 2.36 |
| NR_045838 | A730036I17Rik | chr2 | 3433 | 3.07 | 1.25 | 2.48 |
| TCONS_00017802 | XLOC_014412 | chr2 | 773 | 3.05 | 1.02 | 3.26 |
| AK018082 | AK018082 | chr10 | 1442 | 2.97 | 1.40 | 2.36 |
| ENSMUST00000131854 | Atf3 | chr1 | 517 | 2.56 | 1.30 | 2.35 |
| humanlincRNA1516- | humanlincRNA1516 | chr5 | 4164 | 2.55 | 1.11 | 3.45 |
| AK136555 | AK136555 | chr5 | 2377 | 2.53 | 1.11 | 2.49 |
| ENSMUST00000146366 | Gm5602 | chr7 | 1749 | 2.05 | 0.96 | 2.84 |
| ENSMUST00000171569 | Ndrg1 | chr15 | 4049 | 0.44 | 0.99 | 0.42 |
| AK033431 | AK033431 | chr11 | 2386 | 0.42 | 0.90 | 0.30 |
| uc029xqn.1 | FJ541138 | chrY | 535 | 0.37 | 0.93 | 0.38 |

**Supplementary Table 1. 31 differentially expressed lncRNAs under high-fat diet (HFD) conditions in male and ovariectomized female mice.**

HM = HFD Male; LM = low-fat diet (LFD) Male; HF = HFD Female; LF = LFD Female; HO = HFD Ovariectomy; LO = LFD Ovariectomy.

| **Table S2.**  **Supplementary Table 2. Detailed patient information^*, **^**  ^*^ All liver biopsies were assessed and reviewed by a single experienced liver pathologist. MASLD was defined as the presence of >5% macrovesicular steatosis. MASH was diagnosed based on an overall pattern of histological hepatic injury consisting of macrovesicular steatosis, inflammation, and hepatocellular ballooning. We graded steatosis, lobular inflammation, and hepatocellular ballooning according to the MASLD activity score. Fibrosis was assessed according to a 5-point scale proposed by Brunt and modified by Kleiner et al.   \| **Group** \| **ID** \| **Sex** \| **Age** \| **BMI** \| **Steatosis** \| **Steatosis** \| **Lobular** \| **Portal** \| **Ballooning** \| **NAS** \| **NASH** \| **ALT** \| **AST** \| \| --- \| --- \| --- \| --- \| --- \| --- \| --- \| --- \| --- \| --- \| --- \| --- \| --- \| --- \| \| **%** \| **score** \| **inflammation** \| **inflammation** \| \| Normal \| BS150007054 \| male \| 54 \| 24.8 \| <5 \| 0 \| 0 \| 0 \| 0 \| 0 \| 0 \| 22 \| 24 \| \| BS150015088 \| female \| 49 \| 23.9 \| 0 \| 0 \| 0 \| 0 \| 0 \| 0 \| 0 \| 10 \| 17 \| \| MASL \| BS150002544 \| male \| 55 \| 30.7 \| 33-66 \| 2 \| 0 \| 1 \| 0 \| 2 \| 2 \| 40 \| 36 \| \| BS150010700 \| male \| 38 \| 26.5 \| 33-66 \| 2 \| 1 \| 0 \| 1 \| 4 \| 4 \| 52 \| 60 \| \| BS140021480 \| male \| 43 \| 27.5 \| 33-66 \| 2 \| 1 \| 0 \| 0 \| 3 \| 2 \| 38 \| 28 \| \| MASH  (fibrosis 0-2) \| BS150000530 \| male \| 47 \| 32.1 \| >66 \| 3 \| 2 \| 3 \| 1 \| 6 \| 6 \| 97 \| 80 \| \| BS150000427 \| male \| 20 \| 35.1 \| >66 \| 3 \| 1 \| 0 \| 1 \| 5 \| 5 \| 176 \| 88 \| \| BS150000170 \| male \| 72 \| 27.6 \| 33-66 \| 2 \| 2 \| 3 \| 1 \| 5 \| 3 \| 76 \| 71 \|   ^**^ Grading and staging histological features of NAFLD (MASLD) and NASH (MASH)   - 1. NAFLD Activity Score (NAS; 0-8)      1. Steatosis (0: <5%; 1: 5-33%; 2: 33-66%; and 3: >66%)      2. Lobular inflammation, focci per 20x magnification (0: not present; 1: <2; 2-4; and 3: >4)      3. Ballooning (0: not present; 1: few; and 2: prominent ballooning)   2. NAFLD Fibrosis Score (0-4)      1. 1a: delicate zone 3 psf      2. 1b: dense zone 3 psf      3. 1c: portal only      4. 2: zone 3 plus portal or periportal      5. 3: bridging (c-c, c-p and p-p)      6. 4: cirrhosis |
| --- | --- | --- | --- | --- | --- | --- | --- | --- | --- | --- | --- | --- | --- | --- | --- | --- | --- | --- | --- | --- | --- | --- | --- | --- | --- | --- | --- | --- | --- | --- | --- | --- | --- | --- | --- | --- | --- | --- | --- | --- | --- | --- | --- | --- | --- | --- | --- | --- | --- | --- | --- | --- | --- | --- | --- | --- | --- | --- | --- | --- | --- | --- | --- | --- | --- | --- | --- | --- | --- | --- | --- | --- | --- | --- | --- | --- | --- | --- | --- | --- | --- | --- | --- | --- | --- | --- | --- | --- | --- | --- | --- | --- | --- | --- | --- | --- | --- | --- | --- | --- | --- | --- | --- | --- | --- | --- | --- | --- | --- | --- | --- | --- | --- | --- | --- | --- | --- | --- | --- | --- | --- | --- | --- | --- | --- |

Table S3.

| **REAGENT or**  **RESOURCE** | **SOURCE** | | | **IDENTIFIER** | | |
| --- | --- | --- | --- | --- | --- | --- |
| **Antibodies** | | | | | | |
| anti-TNFα | Santa Cruz Biotechnology | | | sc-1351 | | |
| anti-IL-6 | Santa Cruz Biotechnology | | | sc-28343 | | |
| anti-α-SMA | Cell Signaling | | | 19245S | | |
| anti-WEE1 | Novus Biologicals | | | NBP1-33506 | | |
| anti-p-CDK1 (Y15) | Cell Signaling | | | 10A11 | | |
| anti-p-CDK1 (T161) | Cell Signaling | | | 9114 | | |
| anti-CDK1 | Cell Signaling | | | 28439S | | |
| anti-RNA polymerase II | Abcam | | | ab817 | | |
| anti-TFⅡB | Santa Cruz Biotechnology | | | sc-271736 | | |
| anti-histone 3  (tri methyl K9) | Abcam | | | ab8898 | | |
| anti-histone 3  (tri methyl K4) | Abcam | | | ab8580 | | |
| anti-BNIP3 | Abcam | | | ab109362 | | |
| anti-CYCLIN B1 | Santa Cruz Biotechnology | | | sc-245 | | |
| anti-CYCLIN D | Santa Cruz Biotechnology | | | sc-56302 | | |
| anti-CYCLIN E | Santa Cruz Biotechnology | | | sc-377100 | | |
| anti-Ub | Santa Cruz Biotechnology | | | sc-8017 | | |
| anti-Myc | Santa Cruz Biotechnology | | | sc-40 | | |
| anti-COX4 | Invitrogen | | | 45-8099 | | |
| anti-Phospho-serine/threonine | PhosphoSolutions | | | PM3801 | | |
| anti-ATP5A | Invitrogen | | | 45-8099 | | |
| anti-SIRT3 | Millipore | | | 07-1596 | | |
| anti-SOD2 | Millipore | | | 06-984 | | |
| anti-OXPHOS | Invitrogen | | | 458099 | | |
| anti-GAPDH | Santa Cruz Biotechnology | | | sc-32233 | | |
| anti-β-ACTIN | Santa Cruz Biotechnology | | | sc-47778 | | |
| anti-HSP60 | Abcam | | | ab45134 | | |
| **Chemicals** | | | | | | |
| Adavosertib | MedChemExpress | | | HY-10993 | | |
| Insulin–transferrin–selenium | | | Thermo Fisher Scientific | | | 41400045 |
| Lipofectamine 2000 | | | Invitrogen | | | 11668019 |
| i-StarTaq^TM^ DNA Polymerase | | | iNtRON Biotechnology | | | 25161 |
| SeqAmp DNA Polymerase | | | Takara Bio | | | 638504 |
| Easy-Blue | | | iNtRON Biotechnology | | | 17061 |
| PMSF | | | Sigma-Aldrich | | | P7626 |
| SYBR Green Master mix | | | Applied Biosystems | | | A25742 |
| MitoTracker Green FM | | | Invitrogen | | | M46750 |
| MitoTracker Red CMXRos | | | Invitrogen | | | M7512 |
| Tetramethylrhodamine methyl  and ethyl esters | | | Invitrogen | | | T668 |
| MitoSOX dye | | | Invitrogen | | | M36008 |
| Actinomycin D | | | Sigma-Aldrich | | | A9415 |
| **Critical commercial assays** | | | | | | |
| GeneRacer^TM^ Kit | | | Invitrogen | | | L1502-01 |
| SMARTer RACE 5'/3' kit | | | Takara Bio | | | 634858 |
| RNAscope^TM^ Multiplex  Fluorescent Reagent Kit v2 | | | ACD Bio | | | 323270 |
| Mitochondria isolation kit | | | Thermo Scientific | | | 89874 |
| MEGAscript T7/SP6  Transcription Kit | | | Invitrogen | | | AM1334 |
| **Software and algorithms** | | | | | | |
| Prism | | Graphpad | | | Version 9 | |
| Phenochart^TM^ | | PerkinElmer | | | Version 2 | |
| LAS-X | | Leica | | | Version 1 | |
| Image J | | NIH | | | https://imagej.nih.gov/ij/ | |
| **Others** | | | | | | |
| HFD | | Research Diets | | | D12492 | |
| LFD | | Research Diets | | | D12450J | |
| Western diet | | Research Diets | | | D12079B | |
| CDAA-HFD | | Research Diets | | | A06071306 | |

**Supplementary Table 3. Reagents, software, and diets used for experiments.**

Table S4.

| **Purpose** | **Gene** | **Accession**  **number** | **Nucleotide sequence** | |
| --- | --- | --- | --- | --- |
| qRT-PCR | *Wee1-AS* |  | Sense  Antisense | 5’-CTACCATCCAGCTGAAACTACC-3’  5’-ACAGACATACTACAGCACACTTAC-3’ |
|  | m*Rps14* | NM_020600.4 | Sense  Antisense | 5’-TTTGGTGTCTGCCACATCTT-3’  5’-GGTCAGCCTTCACCTTCATC-3’ |
|  | *snRNA U1* | NR_004413.2  NR_004411.3 | Sense  Antisense | 5’-CCATGATCACGAAGGTGGTTT-3’  5’-ATGCAGTCGAGTTTCCCGCAT-3’ |
|  | *Neat1* | NR_131212.1  NR_003513.3 | Sense  Antisense | 5’-CTCTTCTGTGGTCTCACTCTTC-3’  5’-CTACATCCTCCACAGGCTTAC-3’ |
|  | *Wee1* | NM_001355058.1  NM_009516.3 | Sense  Antisense | 5’-GTCGCCCGTCAAATCACCTT-3’  5’-GAGCCGGAATCAATAACTCGC-3’ |
|  | 18s rRNA | NR_003278.3  NR_046233.2  NR_146144.1  NR_146151.1  NR_146119.1  NR_146117.1  NR_046235.3  NR_145819.1  NR_145820.1 | Sense  Antisense | 5’-GTAACCCGTTGAACCCCATT-3’  5’-CCATCCAATCGGTAGTAGCG-3’ |
|  | *Lnc106435.1* |  | Sense  Antisense | 5’-CAACTCCTACTTTGCCACC-3’  5’-AACTCTTCCCTTCCCATCC-3’ |
|  | *RPS14* | NM_001025070.2 | Sense  Antisense | 5’-CTGGATCCTGGCCAACTCG-3’  5’-CCCTTTCGAGGTGCCATTTTT-3’ |
|  | *snRNA U1* | NR_189289.1  NR_189287.1  NR_104078.1  NR_004400.1  NR_104082.1  NR_004426.2  NR_104085.1  NR_015392.1 | Sense  Antisense | 5’-GAGGCTTATCCATTGCACTCC-3’  5’-AAAGCGCGAACGCAGTC-3’ |
|  | *WEE1* | NM_001143976.2  NM_003390.4 | Sense  Antisense | 5’-ATTTCTCTGCGTGGGCAGAAG-3’  5’-CAAAAGGAGATCCTTCAACTCTGC-3’ |
| ChIP | Wee1 promoter |  | Sense  Antisense | 5’-AGTTTGGCTAGCGCACTCTC-3’  5’-CTCACAGCCAGACTTATCCG-3’ |
| Knockdown  (*in vitro*) | ASO-*Wee1-AS* |  | Sense  Antisense | 5’-ACGGAUAAGUCUGGC-3’  5’-GCCAGACUUAUCCGU-3’ |
|  | si-*LNC106435.1* |  | Sense  Antisense | 5’-GCAGAGAGACAGCUUGUACGC-3’  5’-GCGUACAAGCUGUCUCUCUGC-3’ |
|  | ASO-*LNC106435.1* |  | Sense  Antisense | 5’-AGUGGCACAAUCACAGC-3’  5’-GCUGUGAUUGUGCCACU-3’ |
|  | si-*Wee1* |  | Sense  Antisense | 5’-UCUUGCUCUCACAGUCGUA-3’  5’-UACGACUGUGAGAGCAAGA-3’ |
|  | si-*Cdk1* |  | Sense  Antisense | 5’-GAUGUAAACCCAUGAGAAU-3’  5’-AUUCUCAUGGGUUUACAUC-3’ |
| Knockdown  (*in vivo*) | sh*Wee1* |  |  | #1: TCTTGCTCTCACAGTCGTATGT  #2: CTATTCATGGACACAGAAAAGT |
|  | shScramble |  |  | #1: ACCTAAGGTTAAGTCGCCCTCG  #2: CCAACAAGATGAAGAGCACCAA |

**Supplementary Table 4. Oligonucleotide sequences used in the present investigation.**
