# Supplementary figures and images for: LncRNA Wee1-AS coordinates oxidative fatty acid metabolism through the activation of mitochondrial CDK1/CYCLIN B1
Source: Signal Transduct Target Ther. 2026 Jan 10;11:13. doi: 10.1038/s41392-025-02558-4 (PMC12790571; doi:10.1038/s41392-025-02558-4)

Uncropped western blots


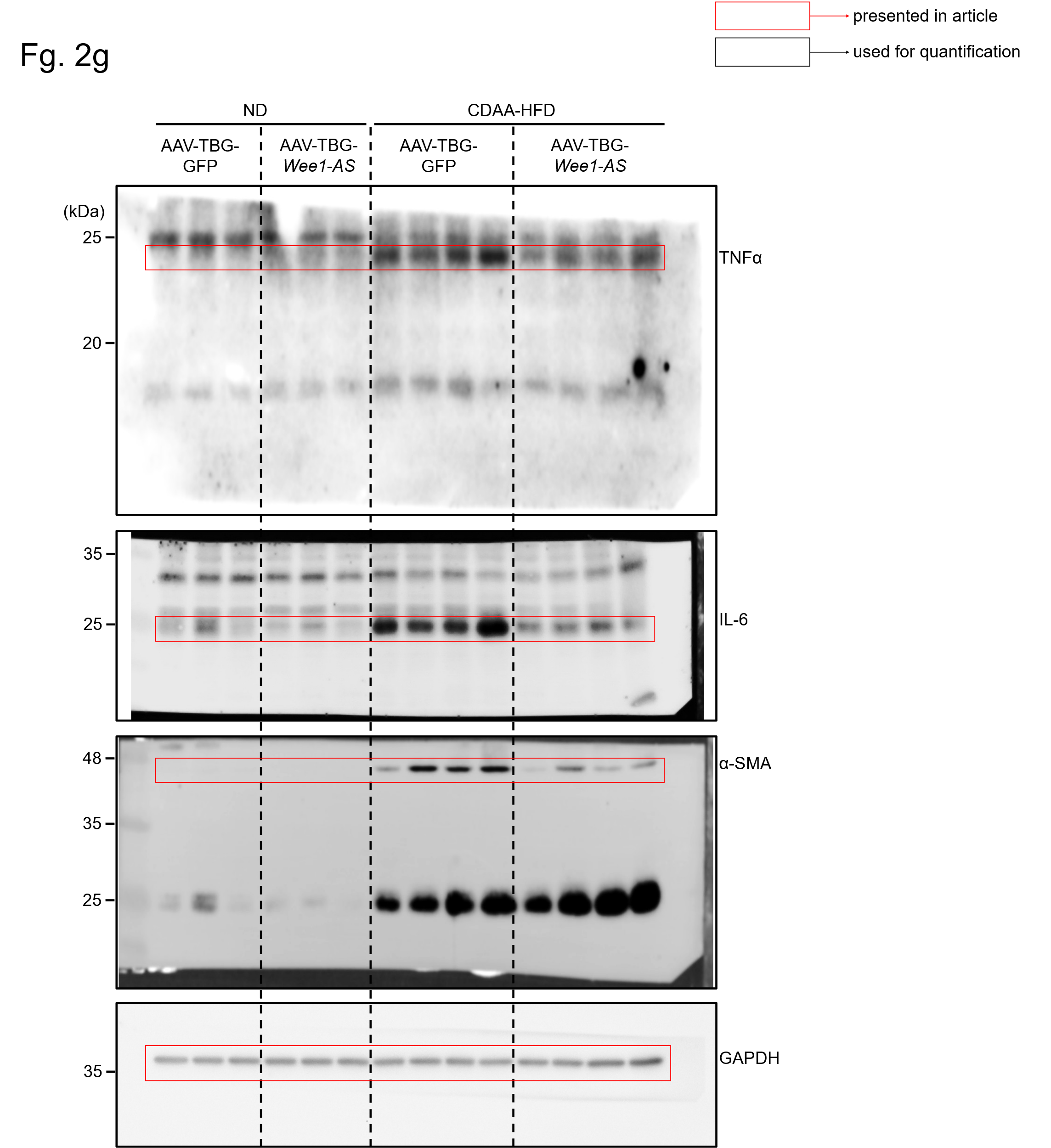


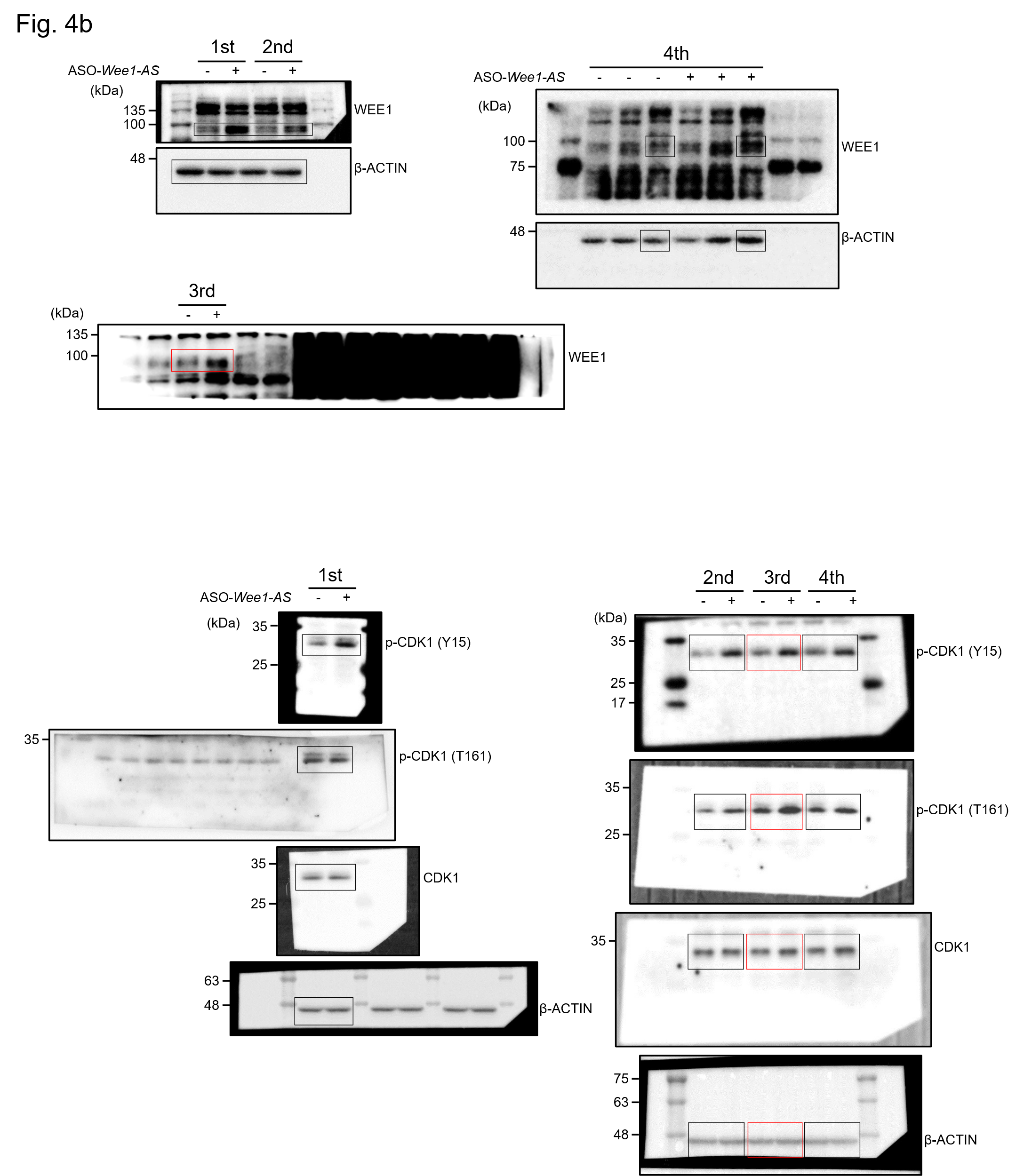


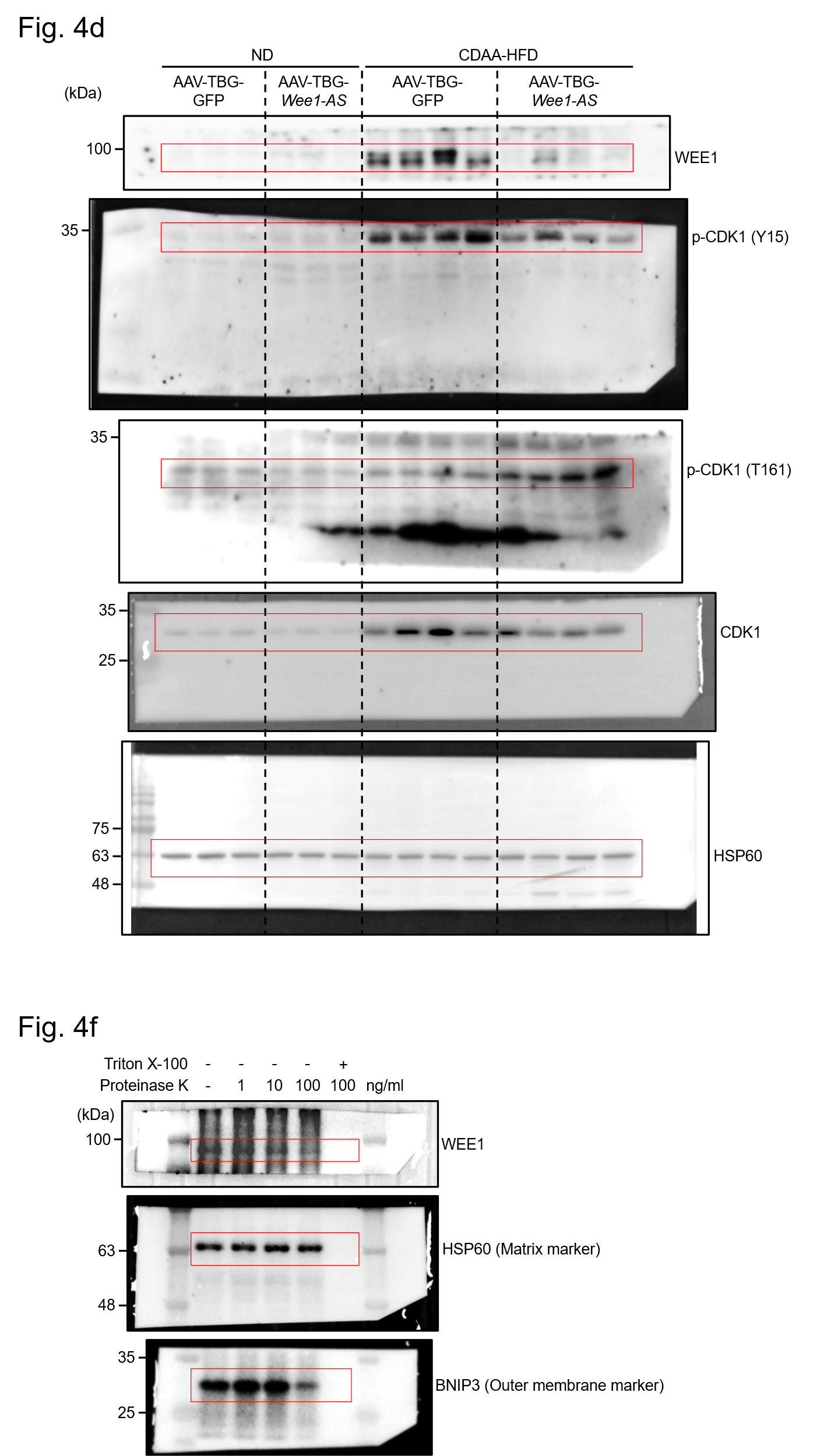


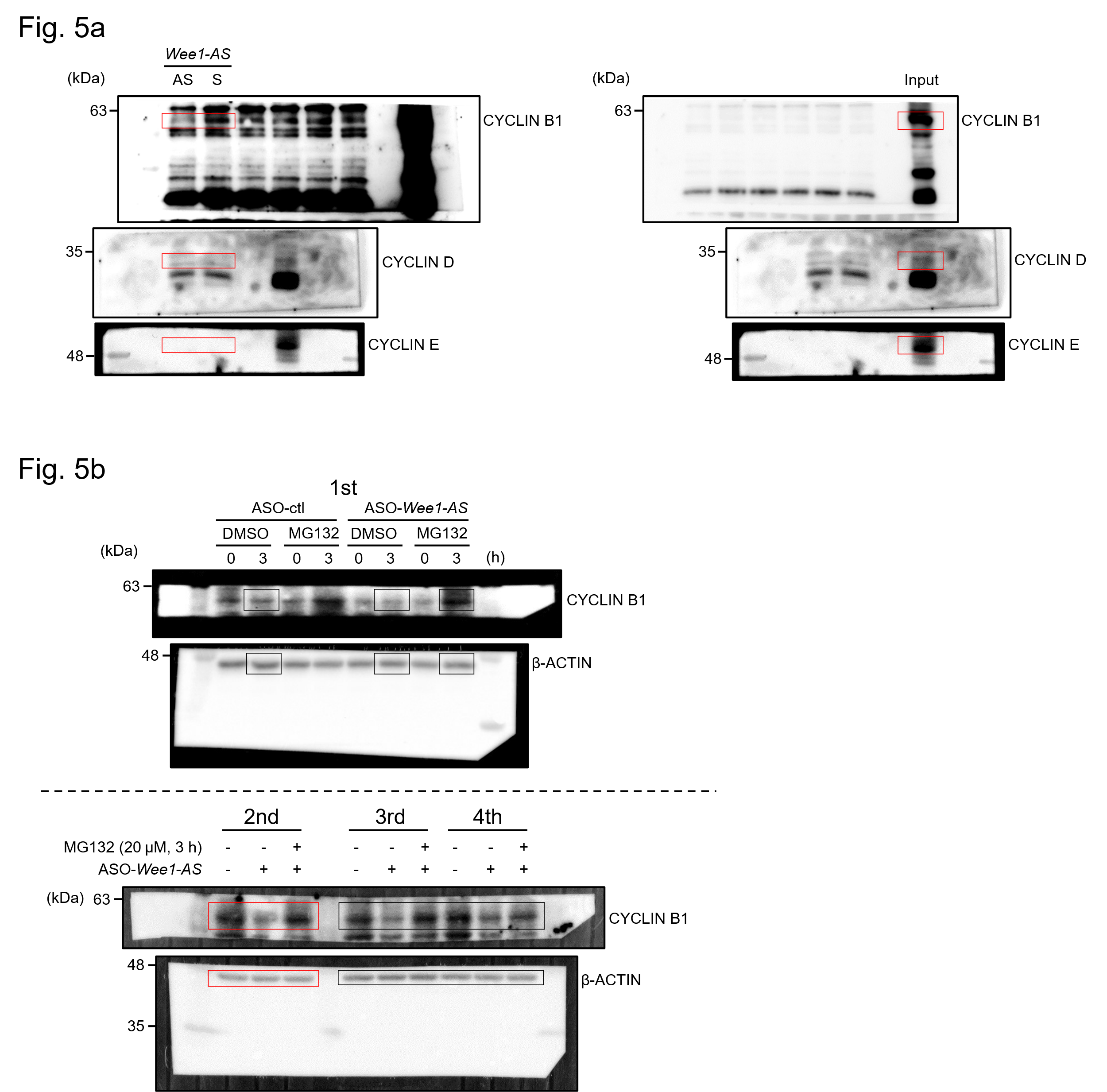


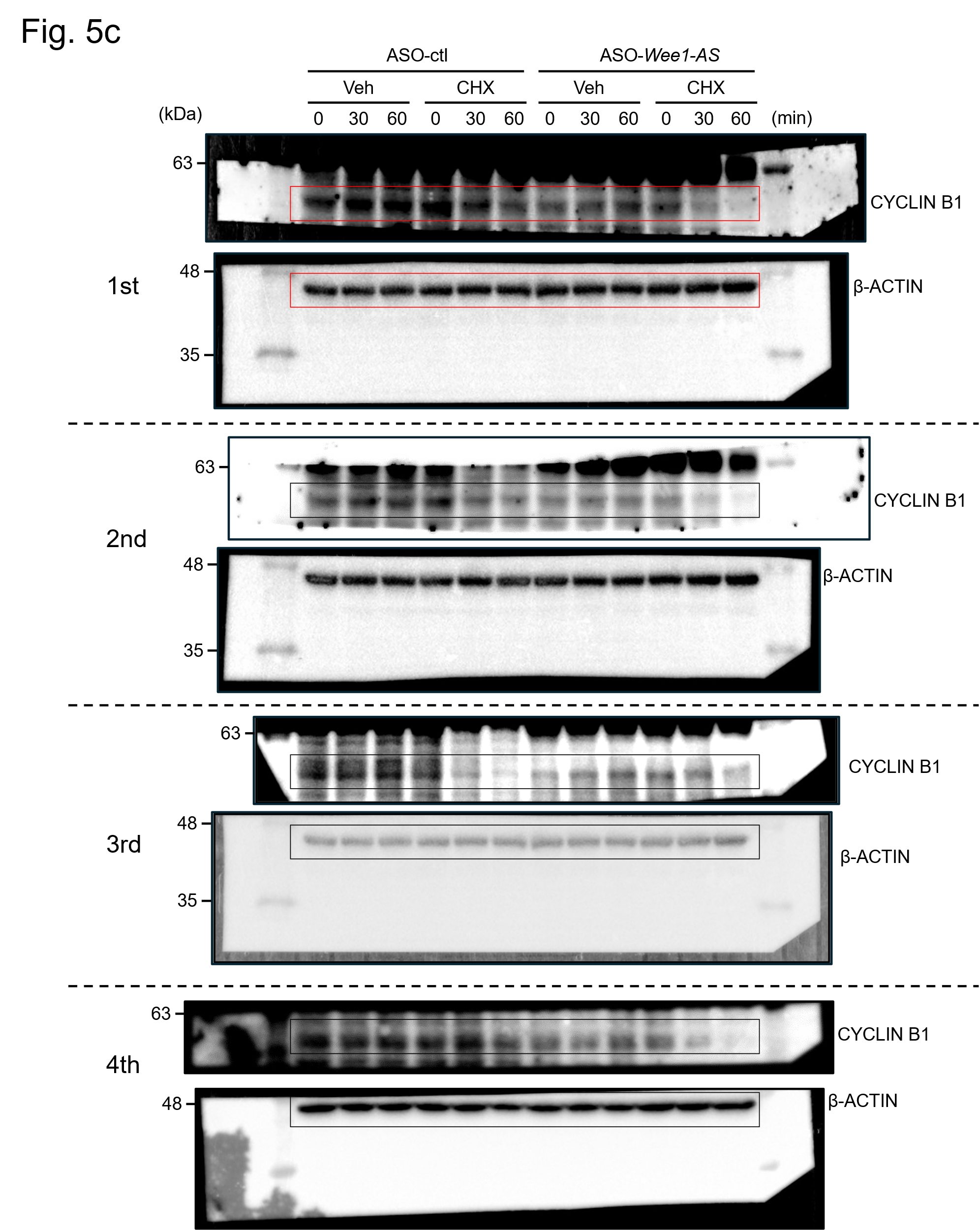


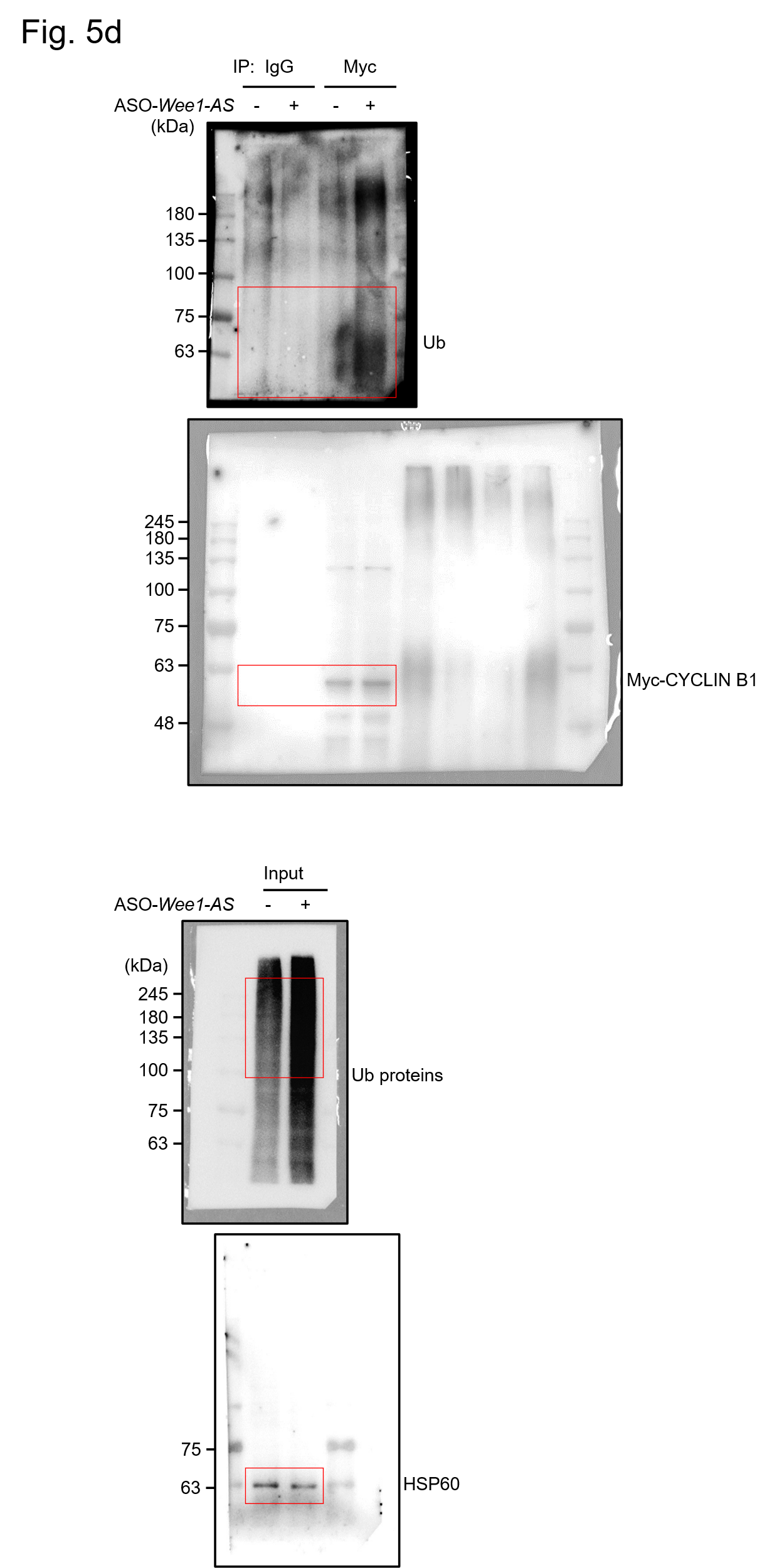


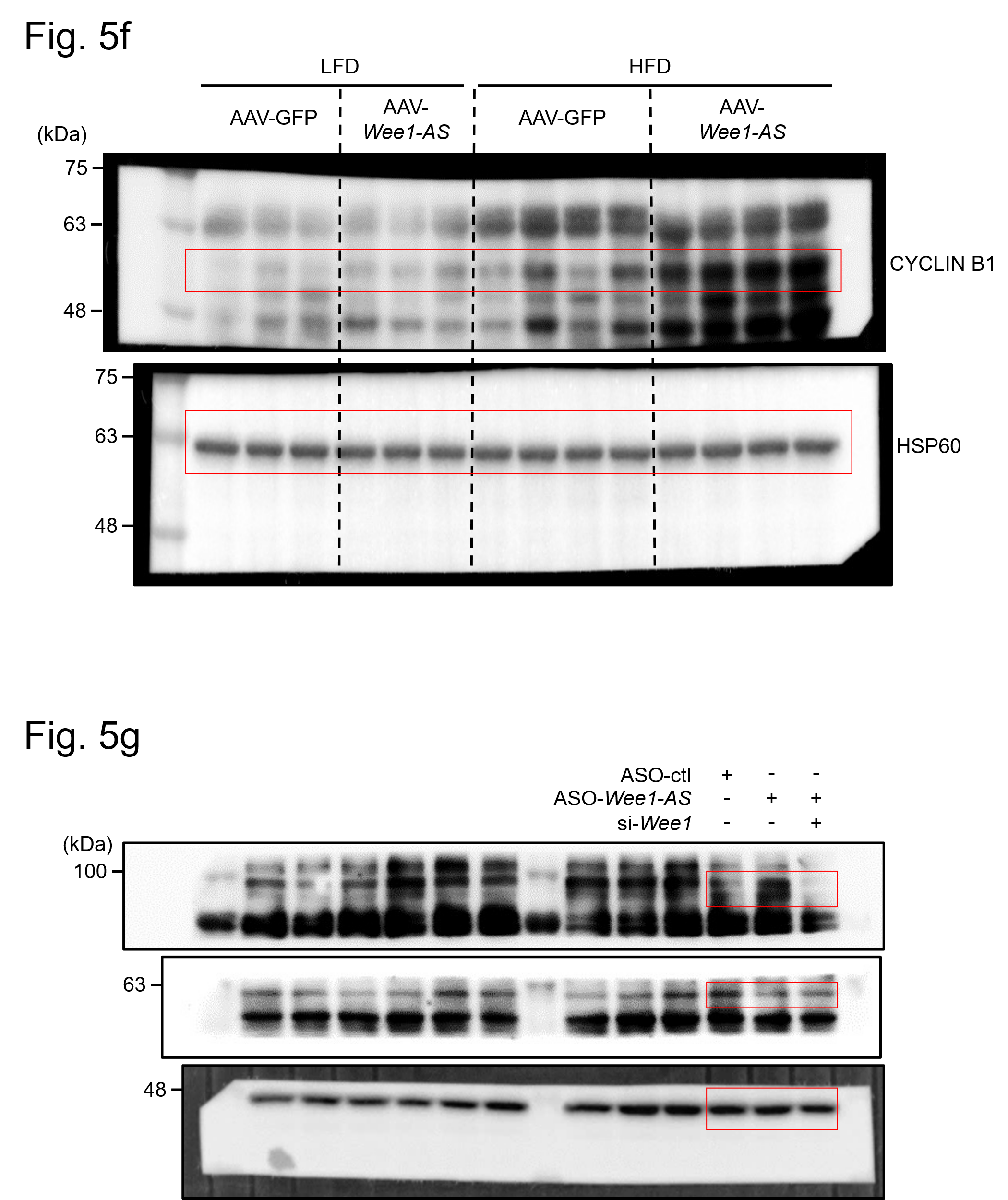


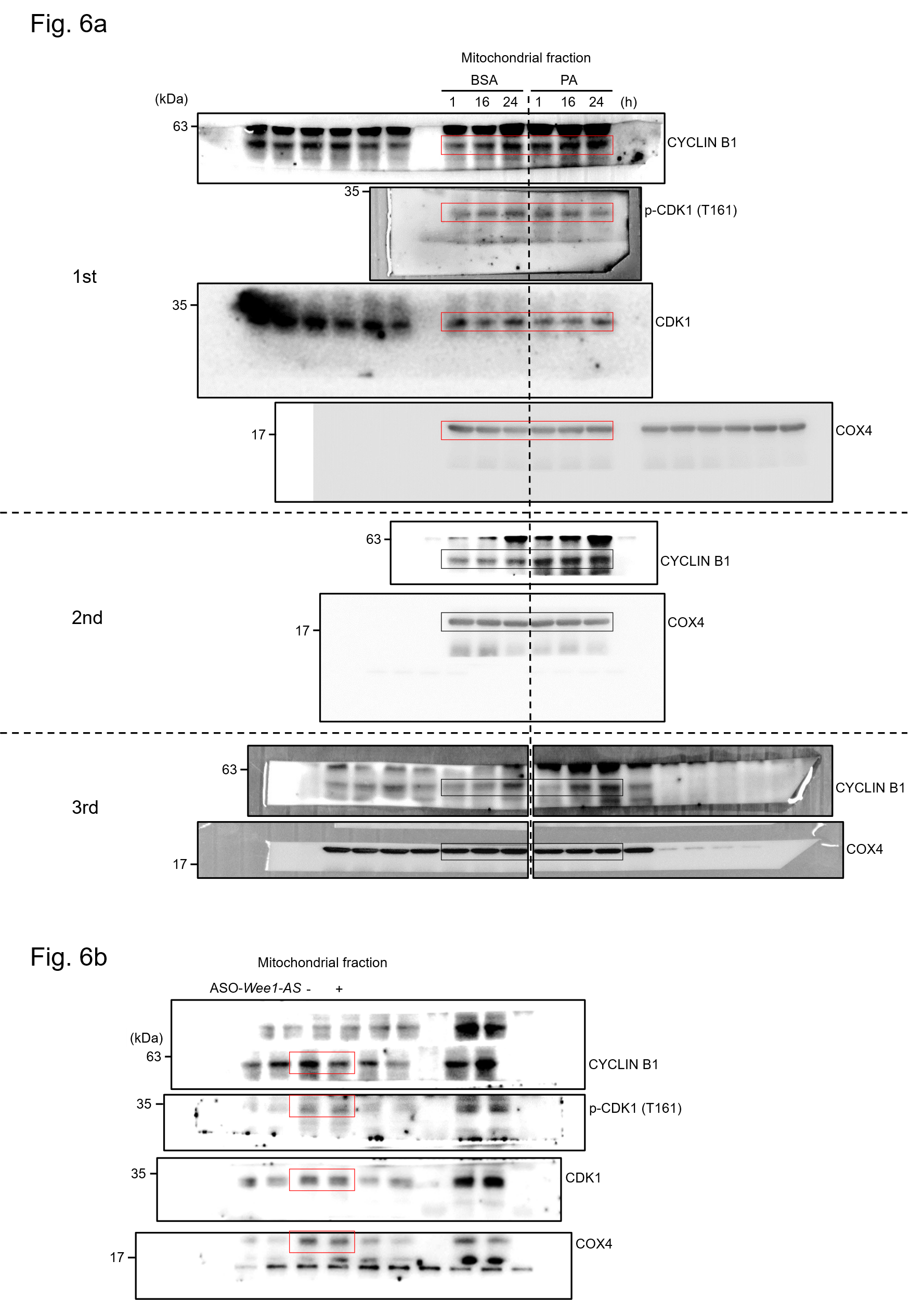


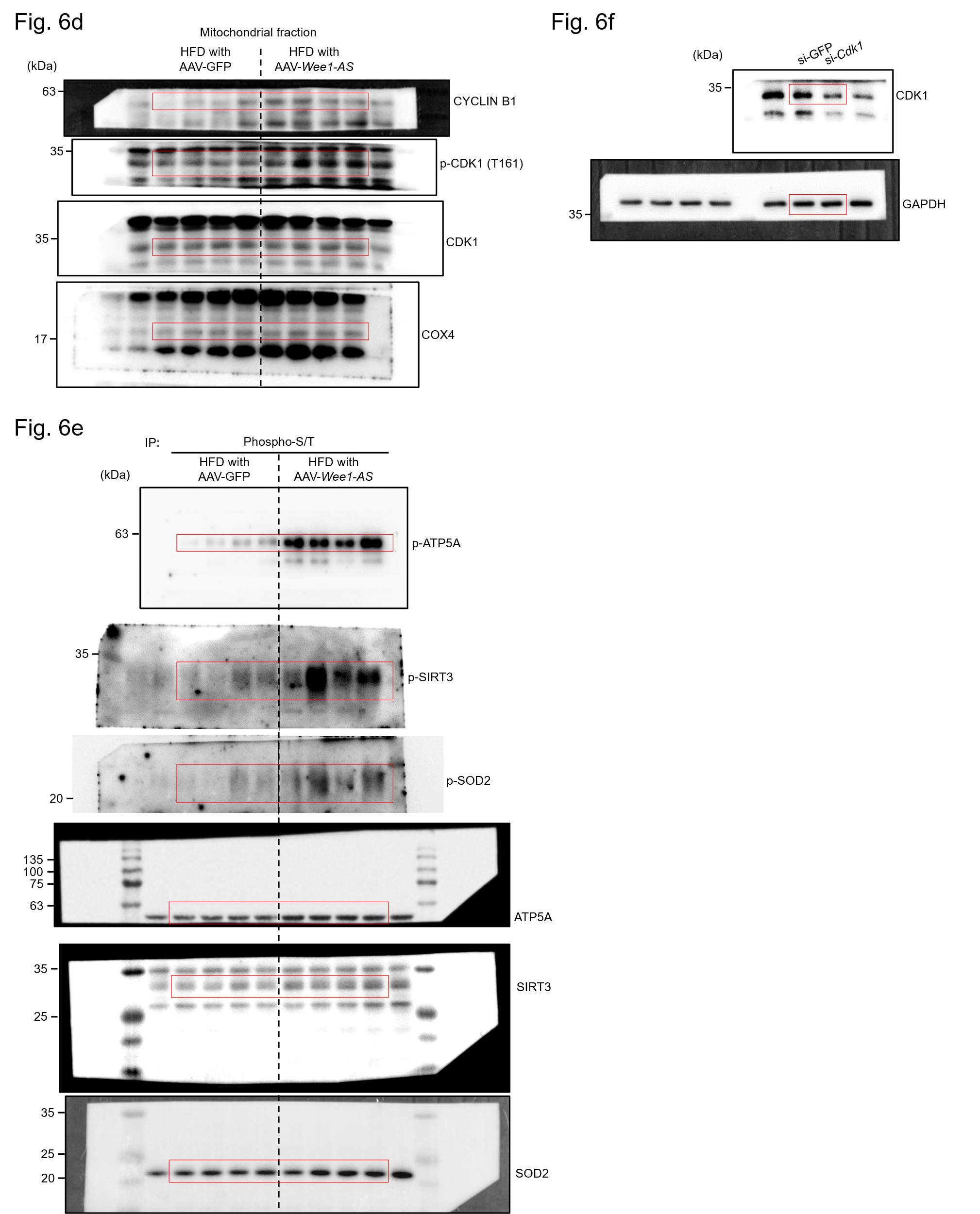


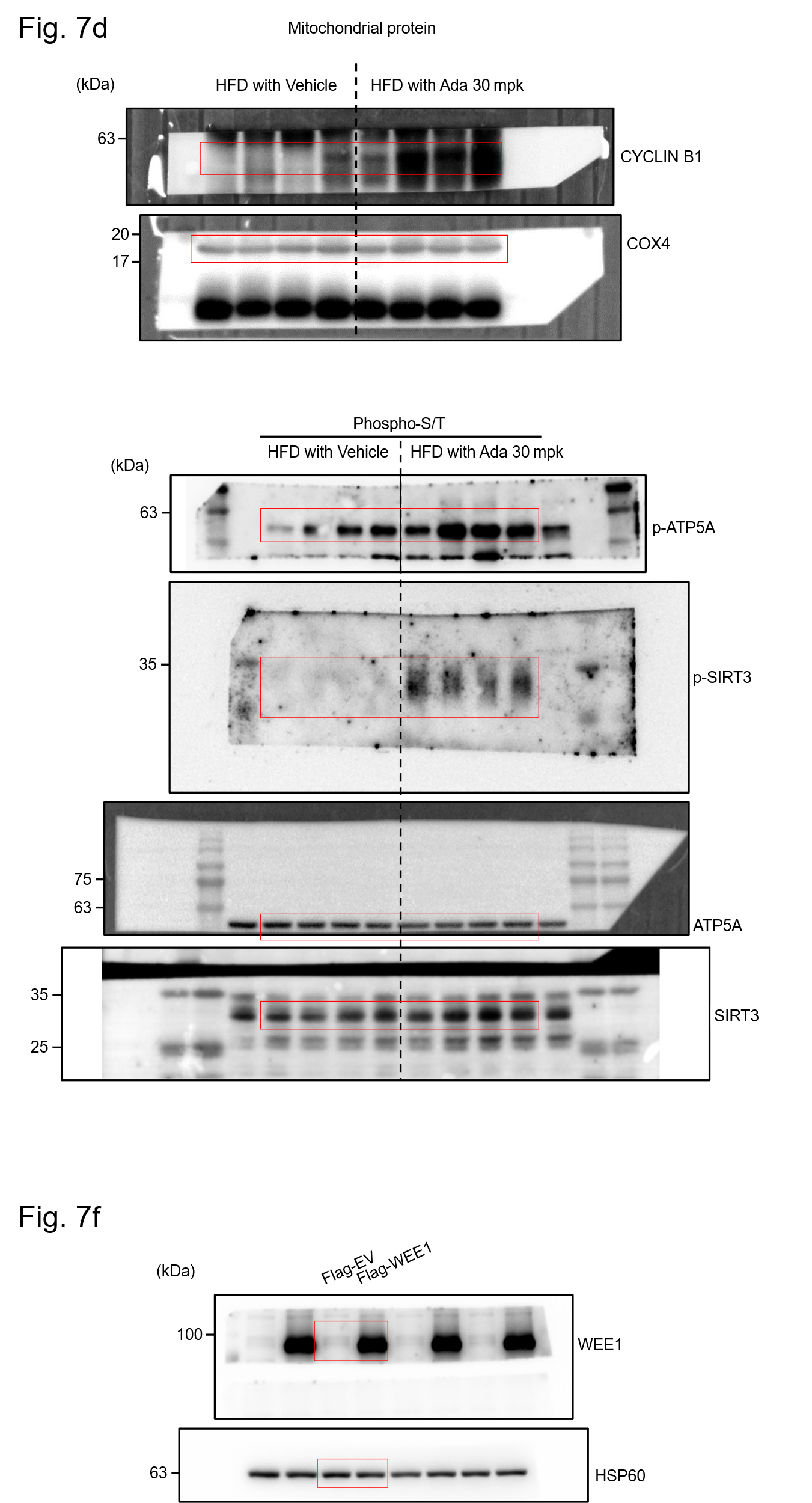


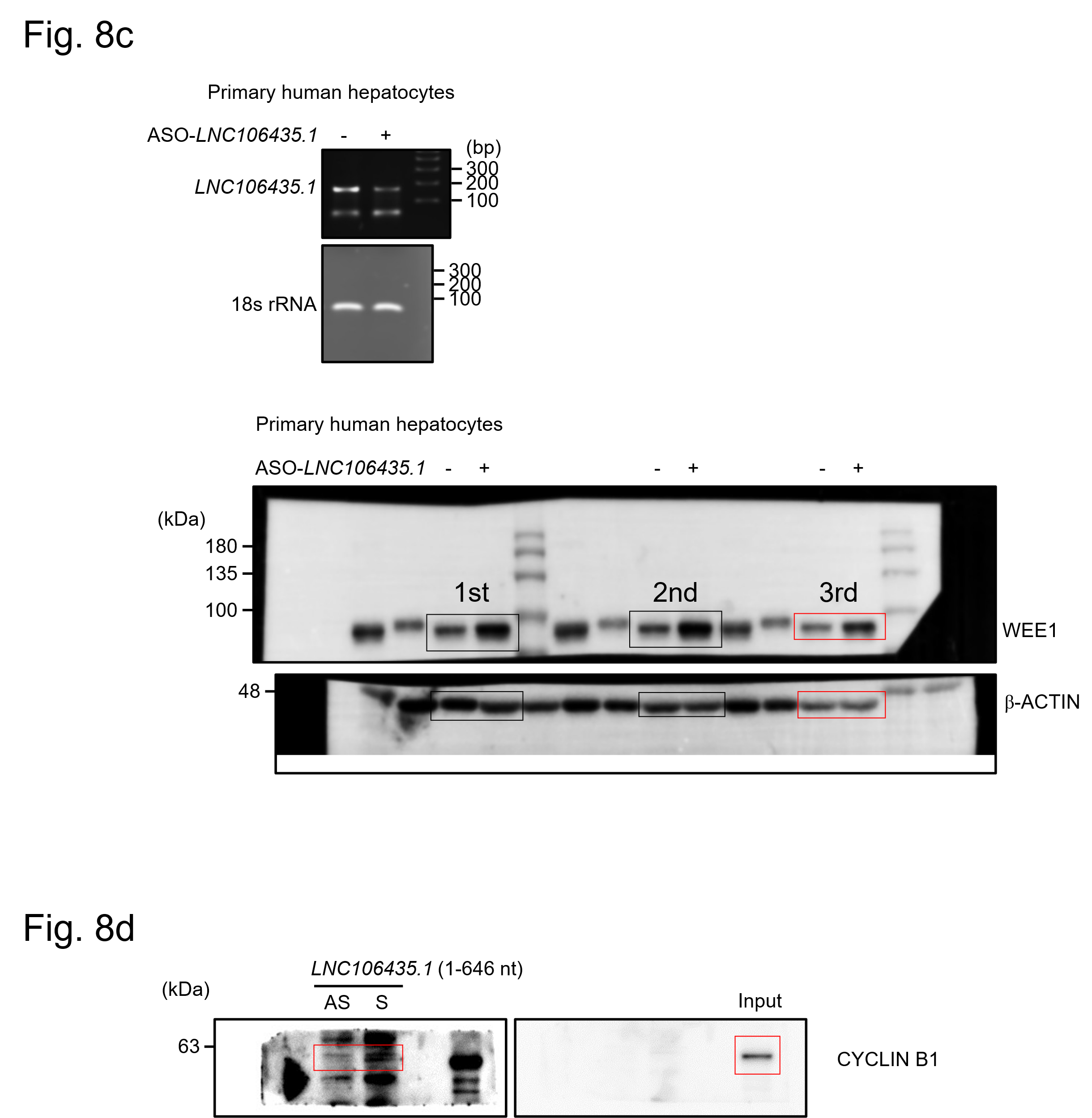


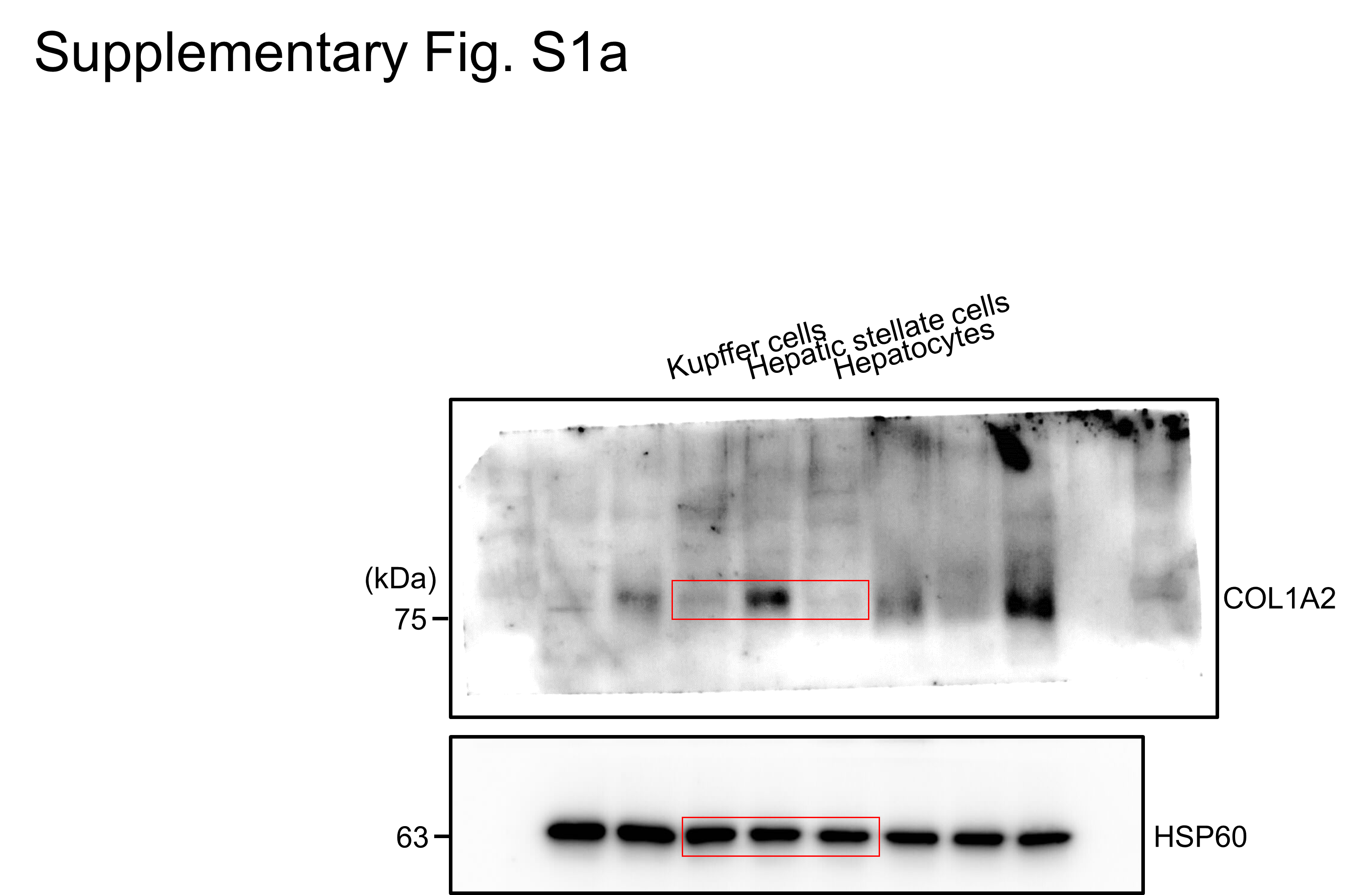


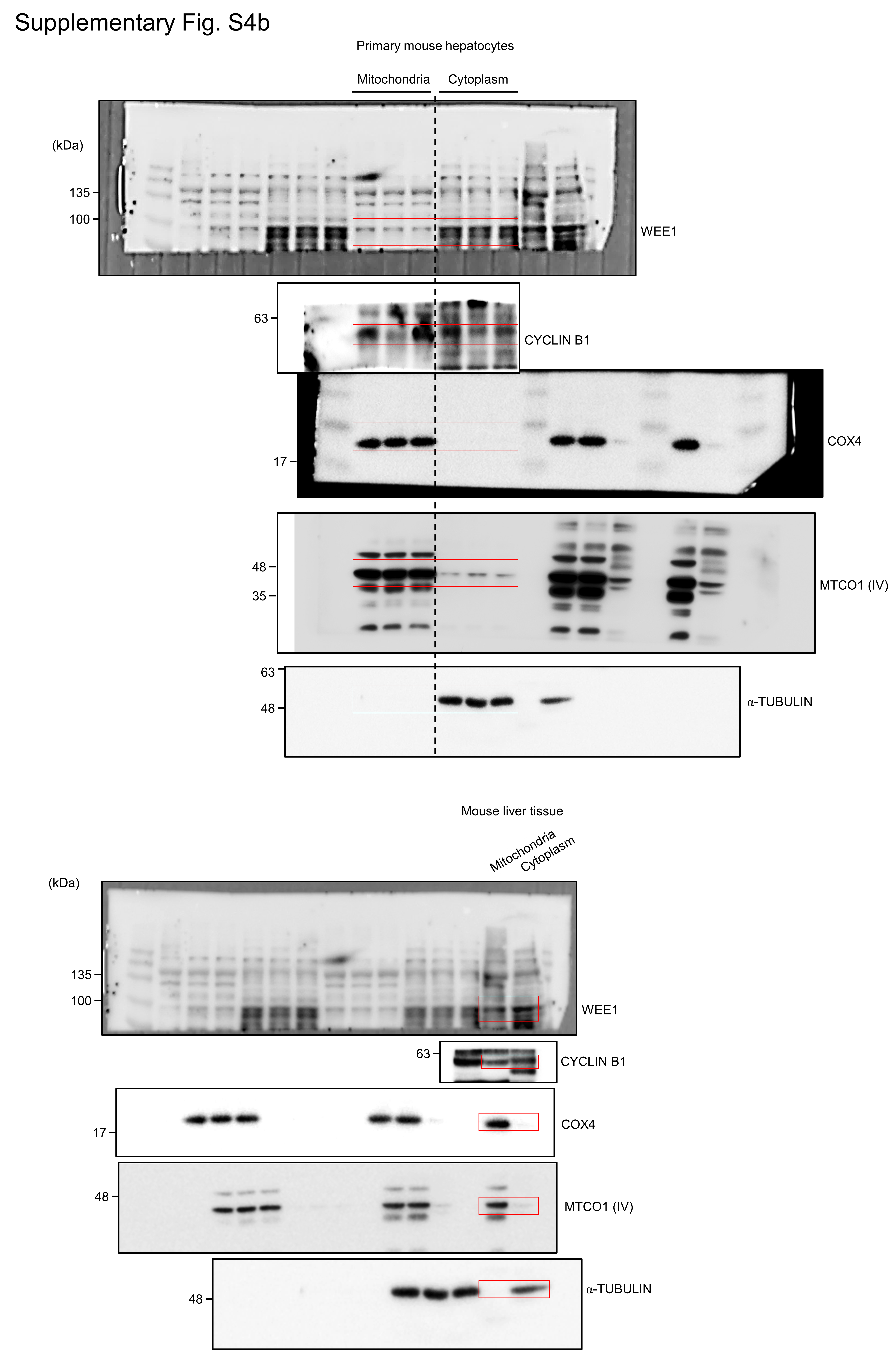


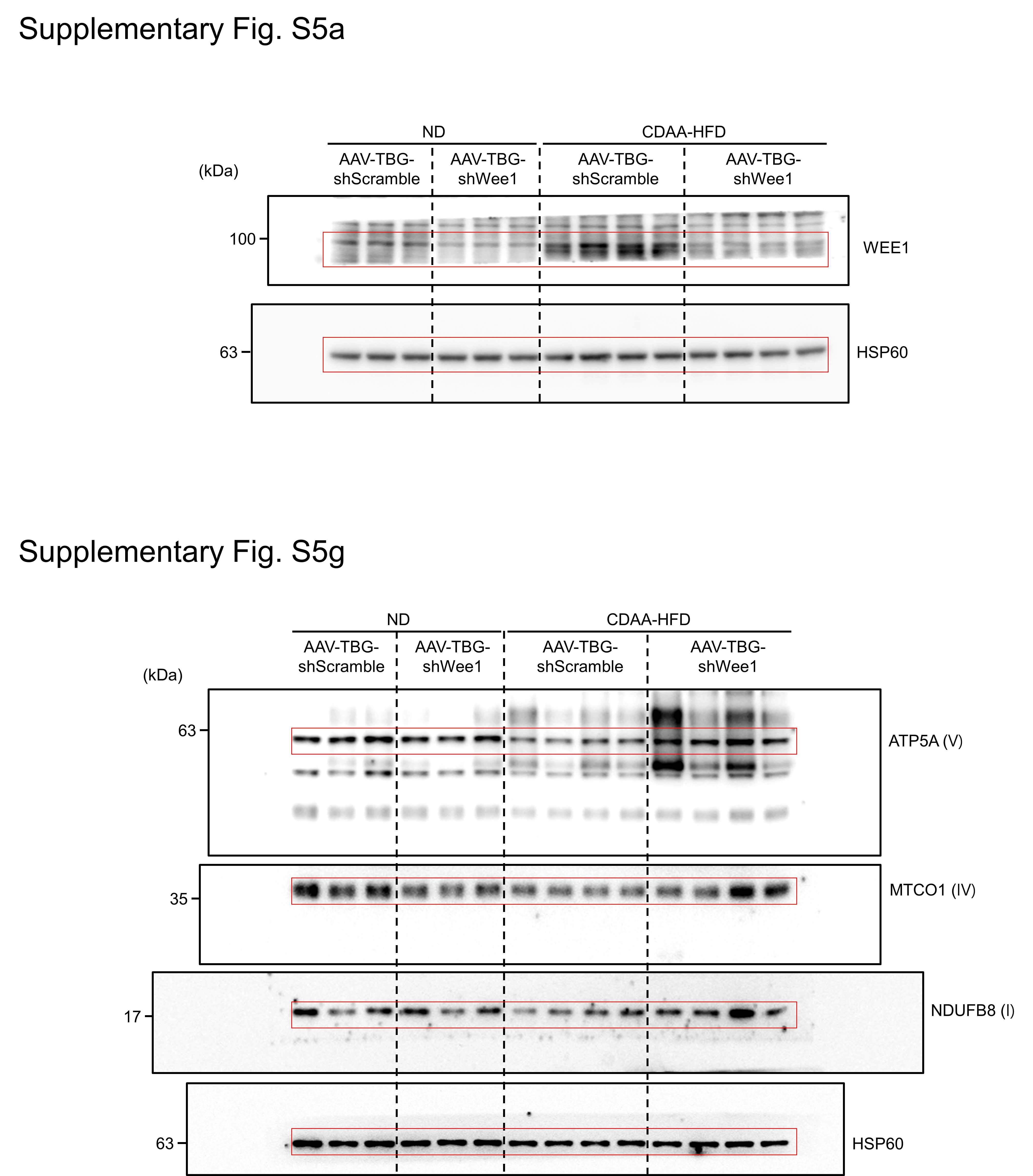


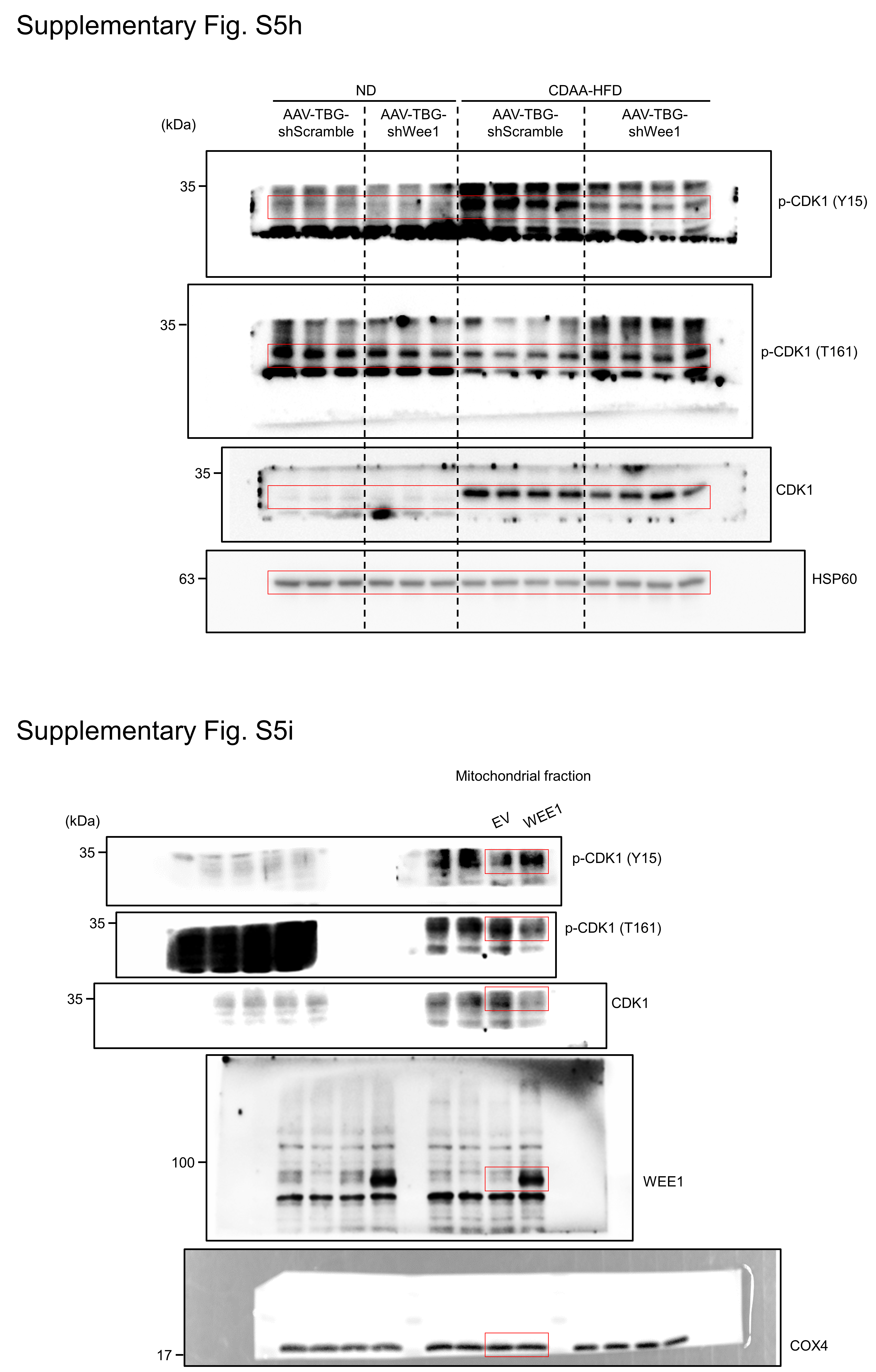


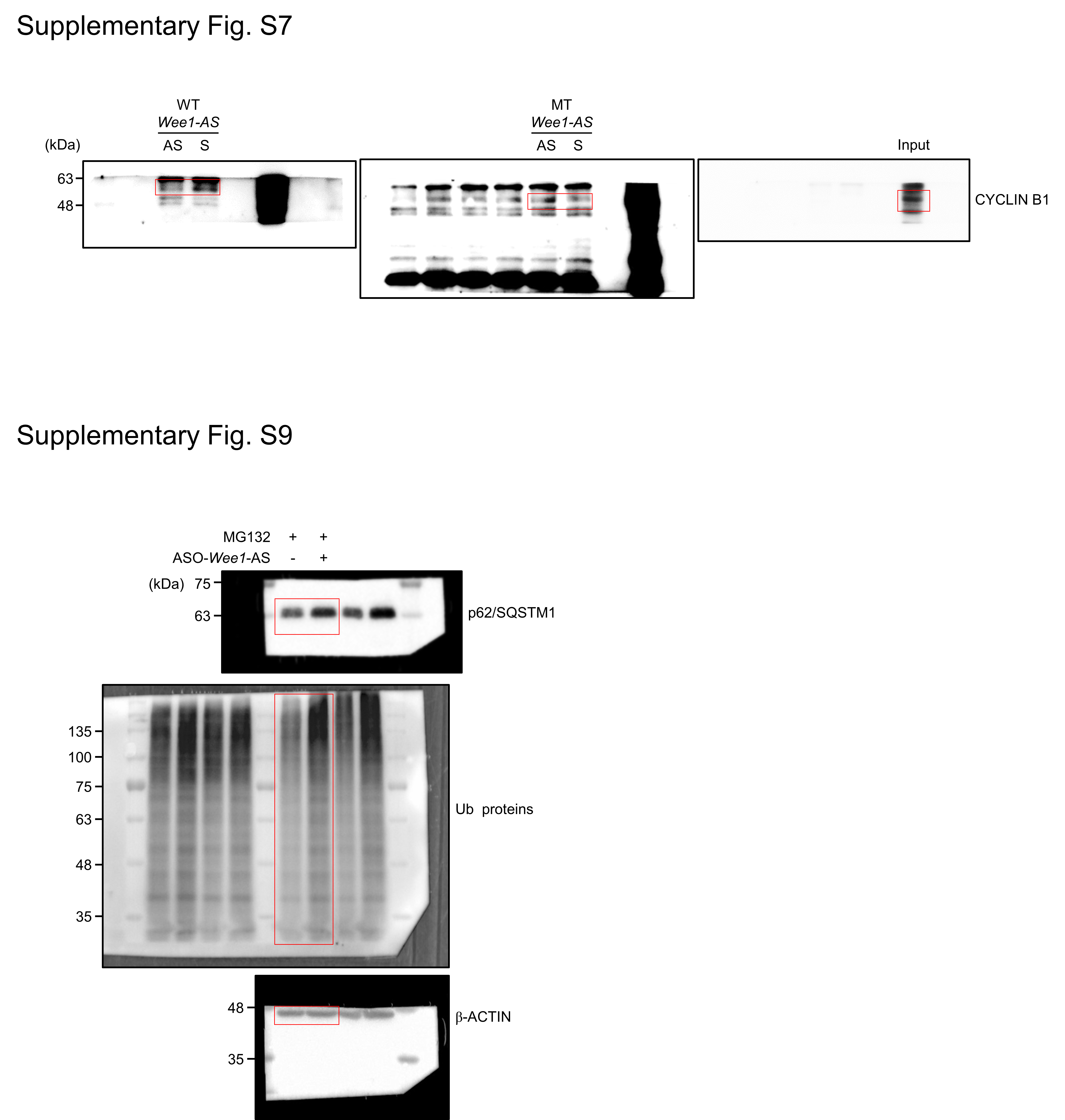

Supplement: Supplementary file 2 — Uncropped western blots [file 41392_2025_2558_MOESM2_ESM.docx]
